# Vimentin filament organization and stress sensing depend on its single cysteine residue and zinc binding

**DOI:** 10.1038/ncomms8287

**Published:** 2015-06-02

**Authors:** Dolores Pérez-Sala, Clara L. Oeste, Alma E. Martínez, M. Jesús Carrasco, Beatriz Garzón, F. Javier Cañada

**Affiliations:** 1 Department of Chemical and Physical Biology, Centro de Investigaciones Biológicas, Consejo Superior de Investigaciones Científicas (C.S.I.C.), Ramiro de Maeztu, 9, 28040 Madrid, Spain

## Abstract

The vimentin filament network plays a key role in cell architecture and signalling, as well as in epithelial–mesenchymal transition. Vimentin C328 is targeted by various oxidative modifications, but its role in vimentin organization is not known. Here we show that C328 is essential for vimentin network reorganization in response to oxidants and electrophiles, and is required for optimal vimentin performance in network expansion, lysosomal distribution and aggresome formation. C328 may fulfil these roles through interaction with zinc. *In vitro*, micromolar zinc protects vimentin from iodoacetamide modification and elicits vimentin polymerization into optically detectable structures; in cells, zinc closely associates with vimentin and its depletion causes reversible filament disassembly. Finally, zinc transport-deficient human fibroblasts show increased vimentin solubility and susceptibility to disruption, which are restored by zinc supplementation. These results unveil a critical role of C328 in vimentin organization and open new perspectives for the regulation of intermediate filaments by zinc.

## Abstract (web summary)

Vimentin is an intermediate filament protein that is targeted by oxidative modification at its single cysteine residue, C328. Here Pérez-Sala *et al*. reveal a role for this cysteine in vimentin organization and function, and uncover a protective role for zinc binding in filament stability.

## Graphical abstract

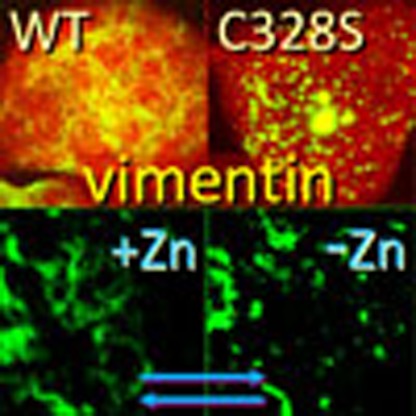

Vimentin is a structural protein belonging to the type III of intermediate filament proteins^1^. It is expressed in cells of mesenchymal origin where it forms the vimentin cytoskeletal network, composed of robust filaments that extend from the nuclear periphery to the cell membrane, conferring mechanical resistance to cells. Although this architectural role was long considered the main function of vimentin, it is now clear that the vimentin network is key for organelle positioning^2^, cell migration and adhesion^3^,^4^, and cell signalling^5^,^6^. Vimentin filaments interact with signalling proteins, such as PLA_2_ and 14-3-3 proteins. In addition, vimentin binds to phosphorylated ERK^7^ and to RhoK^1^, modulating MAPK cascades and potentially actin organization.

Vimentin also has critical implications in pathophysiology. Previously considered as a marker for epithelial–mesenchymal transition, it is now recognized as an active factor in this process, and therefore in cancer cell dissemination^8^,^9^. Vimentin is essential in wound healing, but it can also promote excessive scarring^10^,^11^,^12^. Moreover, it plays a facilitating role in certain viral infections^13^,^14^ and as an autoantigen in the pathogenesis of rheumatic fever^15^ and rheumatoid arthritis^16^.

Structurally, vimentin is a coiled-coil protein composed mainly of α-helical regions linked by connecting segments. Although several vimentin segments and partial oligomers have been crystallized^17^, a crystal structure for full-length monomeric or tetrameric vimentin is not yet available. Molecular modelling studies^18^,^19^,^20^ propose that vimentin monomers assemble into parallel dimers that in turn associate in an antiparallel, staggered manner into tetramers, which are considered as the structural units for vimentin polymerization. Crosslinking studies have shown that several modes of assembly are possible for vimentin tetramers^21^,^22^. Eight tetramers would assemble into ‘unit-length filaments'^23^,^24^ that connect head to tail and compact to give the 10-nm-wide mature filaments^1^. This process can be elicited *in vitro* by increasing the ionic strength.

In cells, it is considered that vimentin particles fuse into elongated structures known as squiggles that assemble into filaments^25^. Contrary to polymerization of microtubules and microfilaments, intermediate filament polymerization is nonpolar and may occur at either end of the filament, and individual subunits can be exchanged anywhere along the filament length^26^,^27^,^28^. The cellular mechanisms regulating the vastly dynamic vimentin network include interactions with scaffold and cytoskeletal proteins^4^, in particular, microtubules and their associated molecular motors (see refs 1, 29 for review). Conversely, vimentin phosphorylation at specific sites by various kinases controls filament disassembly^30^. In addition, vimentin is the target for various non-enzymatic modifications, mostly oxidative in nature, including glutathionylation, nitrosylation or carbonylation^31^,^32^,^33^,^34^, which may be involved in the regulation of the vimentin network under stress conditions.

Cyclopentenone prostaglandins (cyPG) are reactive lipids that are generated in increased levels under conditions of inflammation or oxidative stress^35^,^36^. cyPG play important roles in the regulation of cell proliferation, inflammation and the stress response through their ability to covalently modify signalling proteins, transcription factors and their regulators at specific cysteine residues^37^,^38^,^39^. We previously showed that cyPG bind covalently to vimentin at its single cysteine residue (C328) and cause a rearrangement of the vimentin network^40^,^41^.

Here we have addressed the importance of C328 both in vimentin organization and response to various types of electrophilic and oxidative stress. Our results show that C328 is required for the correct function of vimentin under resting conditions and for its plasticity in response to oxidative stress. Moreover, we show that this dual function of vimentin C328 is related to its ability to respond to the modulation of zinc levels, which regulate vimentin polymerization and C328 modification both in cells and *in vitro*, thus suggesting that this cysteine residue participates in zinc binding. These findings shed new light on the biology of vimentin organization with implications for the regulation of intermediate filament function.

## Results

### Vimentin is the target for various electrophilic lipids

**Figure 1:**
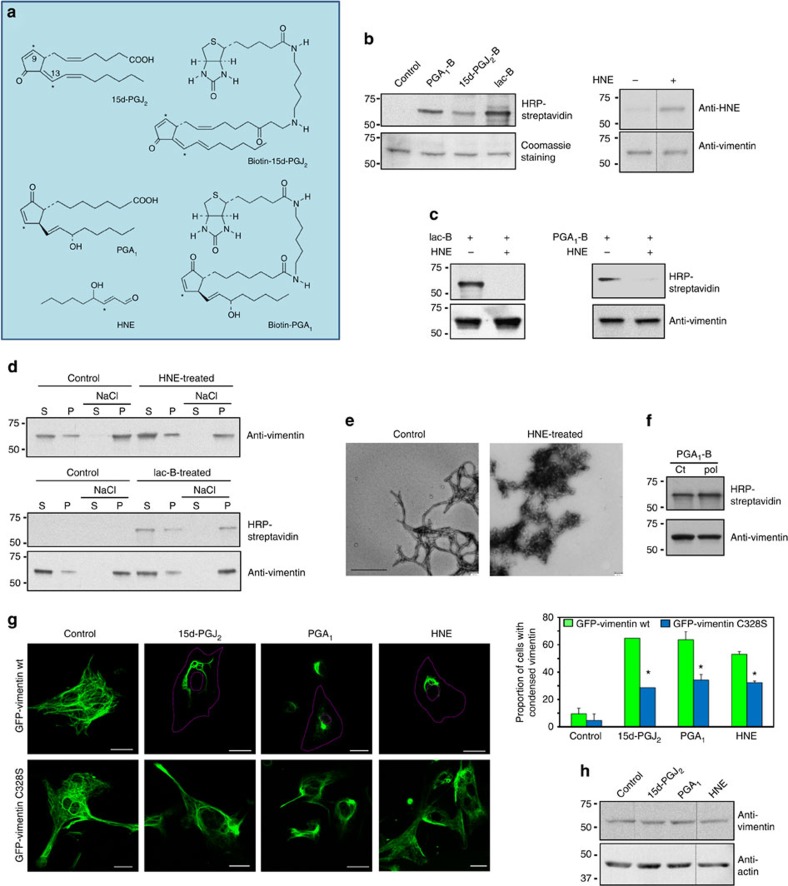
Modification of vimentin by various electrophiles. (**a**) Structures of the electrophilic lipids used. Asterisks denote carbons susceptible of Michael addition. (**b**) Vimentin was incubated with vehicle, PGA_1_-B, 15d-PGJ_2_-B or Iac-B at 10 μM, or 50 μM, HNE for 2 h at 37 °C. Incorporation of electrophiles as well as vimentin levels were assessed by WB. The position of molecular weight (MW) standards (in kDa) is indicated on the left of the panels. Dividing lines indicate where lanes from the same gel have been cropped. (**c**) Competition between various electrophiles for binding to vimentin. Vimentin was preincubated with 100 μM HNE for 2 h at 37 °C, after which, 10 μM Iac-B or 1 μM PGA_1_-B were added and incubation was continued for 30 min at r.t. (**d**) Effect of vimentin modification by electrophiles on polymerization. Vimentin was incubated with vehicle (control), 50 μM HNE (upper panel) or 100 μM Iac-B (lower panel) for 2 h at 37 °C, polymerized with 150 mM NaCl, and soluble (S) and polymerized (P) vimentin were separated by ultracentrifugation. Incorporation of Iac-B into vimentin was assessed as above. (**e**) Electron microscopy (EM) of vimentin filaments formed before or after treatment with HNE. Vimentin was incubated in the absence or presence of HNE as in **d**, before polymerization with 150 mM NaCl and visualization by EM. Scale bar, 500 nm. (**f**) Incorporation of PGA_1_-B into soluble or polymerized vimentin. Soluble (ct) or polymerized (pol) vimentin was incubated with 10 μM PGA_1_-B and its binding to vimentin was assessed as above; *n*≥3 for all blots. (**g**) Effect of various electrophiles on the vimentin cytoskeleton in RMC. RMCs were transfected with GFP-vimentin wt or C328S and treated 48 h later with 7.5 μM 15d-PGJ_2_, 30 μM PGA_1_ or 10 μM HNE for 4 h and observed live by confocal microscopy. Scale bars, 20 μm. The proportion of cells with condensed vimentin was estimated, right panel (mean±s.e.m. of three independent experiments, *P<0.05 by Student's *t*-test). (**h**) RMC treated with 15d-PGJ_2_ or PGA_1_ for 22 h or with HNE for 4 h were lysed and vimentin levels assessed by WB.

Vimentin is the target for several thiol-reactive compounds that alter the organization of its network^34^,^42^. To characterize the mechanisms involved, we first explored the binding of several electrophiles (Fig. 1a) to vimentin by gel-based *in vitro* assays. Biotinylated derivatives of the cyPG PGA_1_, 15d-PGJ_2_ and iodoacetamide (Iac), as well as the reactive aldehyde 4-hydroxynonenal (HNE) covalently bound to vimentin (Fig. 1b). Incubation with HNE precluded vimentin modification by PGA_1_-B and biotinylated Iac (Iac-B; Fig. 1c), pointing to vimentin's C328 as a common site for electrophile addition. Incubation with HNE or Iac-B did not block vimentin polymerization (Fig. 1d), but severely altered filament morphology, yielding ‘garland-like' structures (Fig. 1e). Conversely, prior polymerization of vimentin did not preclude electrophile addition, as shown here for PGA_1_-B (Fig. 1f). We then explored the effect of various electrophilic compounds on vimentin organization using rat mesangial cells (RMC), in which green fluorescent protein-tagged vimentin (GFP-vimentin) incorporates into the endogenous network (Fig. 1g). Treatment of GFP-vimentin wild-type (wt)-transfected cells with 15d-PGJ_2_, PGA_1_ or HNE induced a marked perinuclear condensation of intermediate filaments (Fig. 1g), without detectably altering vimentin levels or integrity (Fig. 1h). Interestingly, in GFP-vimentin C328S-transfected cells, the effect of electrophiles was attenuated (Fig. 1g), as evidenced by a lower proportion of cells showing full perinuclear vimentin condensation (Fig. 1g, graph). This suggests that C328 is important for rearrangement of the vimentin network induced by electrophilic agents.

### C328 of vimentin plays a key role in filament formation

**Figure 2:**
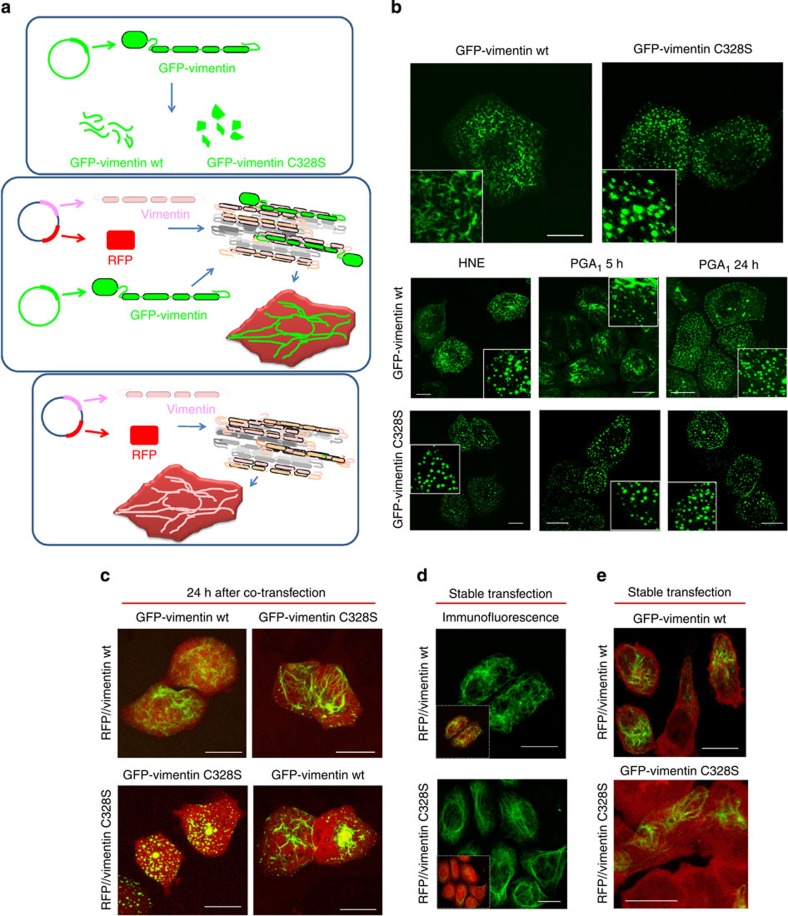
Strategies for the expression and distribution of vimentin constructs in vimentin-deficient SW13 cells. (**a**) Plasmids used for different transfection strategies in SW13 cells. Upper panel, stable transfection with GFP-vimentin fusion constructs gives rise to squiggles (in the case of GFP-vimentin wt) or to dots (in the case of GFP-vimentin C328S); middle panel, transfection of RFP//vimentin plus GFP-vimentin, allowing assembly of untagged vimentin, detection of transfected cells (RFP positive) and incorporation of GFP-vimentin; lower panel, stable transfection of RFP//vimentin. (**b**) SW13 cells were stably transfected with the indicated constructs. Vimentin distribution was observed by live confocal microscopy. Lower panels, transfected cells were treated with 10 μM HNE for 4 h or with 30 μM PGA_1_ for the indicated times. Insets show enlarged areas of interest. (**c**) Cells were co-transfected with the indicated constructs before live visualization. (**d**) Cells were stably transfected with the bicistronic RFP//vimentin wt or C328S plasmids, and vimentin was visualized by IF. Insets show overlays with RFP. (**e**) Stably RFP//vimentin wt and C328S-expressing cells were transfected with GFP-vimentin fusion constructs and visualized live. *n*≥3 for all assays; scale bars 20 μm.

Confirmation of C328 importance required using a vimentin-deficient cell model to transfect a homogeneous population of vimentin molecules. The adrenal carcinoma cell line SW13/cl.2 (SW13), devoid of cytosolic intermediate filaments^43^, was used for various transfection strategies (Fig. 2a). First, cells were stably transfected with GFP-vimentin wt or C328S (Fig. 2a, upper panel). GFP-vimentin wt-transfected cells showed filamentous structures with two free ends consistent with vimentin squiggels or short vimentin filaments. In sharp contrast, cells transfected with GFP-vimentin C328S showed only a bright punctate pattern consistent with vimentin dots or aggregates^44^. Similar results were obtained with GFP-vimentin C328A (Supplementary Fig. 1). Interestingly, treatment of SW13 cells expressing GFP-vimentin wt with HNE induced filament disassembly, leading to vimentin dots (Fig. 2b, middle panels). Upon treatment with PGA_1_, condensed filaments coexisting with dots were observed after 4–5 h, whereas after 24 h only bright vimentin dots were left. The punctate pattern of GFP-vimentin C328S was not further altered by treatment with these agents (Fig. 2b, lower panels). Thus, in the absence of endogenous vimentin, the GFP molecule imposes a restriction on the formation of full-length filaments that is much more severe in the case of C328S vimentin, unveiling the essential role of C328 for elongation of GFP-vimentin.

Since GFP-vimentin wt or C328S can form a normal network in cells expressing endogenous vimentin (Fig. 1), we co-transfected SW13 cells with bicistronic expression plasmids coding for untagged vimentin wt or C328S and DsRed-Express2 protein (RFP) as separate products (RFP//vimentin constructs), along with GFP-vimentin wt or C328S fusion constructs to ‘light up' the filaments formed (Fig. 2a, middle panel). After 24 h, only cells co-transfected with RFP//vimentin wt plus GFP-vimentin wt (Fig. 2c) showed an extended vimentin network, with cells expressing RFP//vimentin C328S plus GFP-vimentin C328S displaying only dots. These results clearly show that C328 is essential for the formation of the vimentin intermediate filament network at short times after transfection. Interestingly, co-transfection with vimentin wt plasmids partially rescued the defect of vimentin C328S.

To assess the capacity of vimentin wt and C328S to form filaments in the long run, cells were stably transfected with the bicistronic plasmids (RFP//vimentin wt or C328S) (Fig. 2a, lower panel). Vimentin immunofluorescence (IF) showed formation of full filaments by both constructs (Fig. 2d). Similarly, once a vimentin network is formed, transient transfection of these cells with GFP-vimentin wt or C328S allowed the detection of an extended vimentin network in both cases (Fig. 2e). Therefore, vimentin C328S is able to form full-length filaments in cells, but requires more time than vimentin wt.

### C328 affects vimentin network extension and subunit exchange

**Figure 3:**
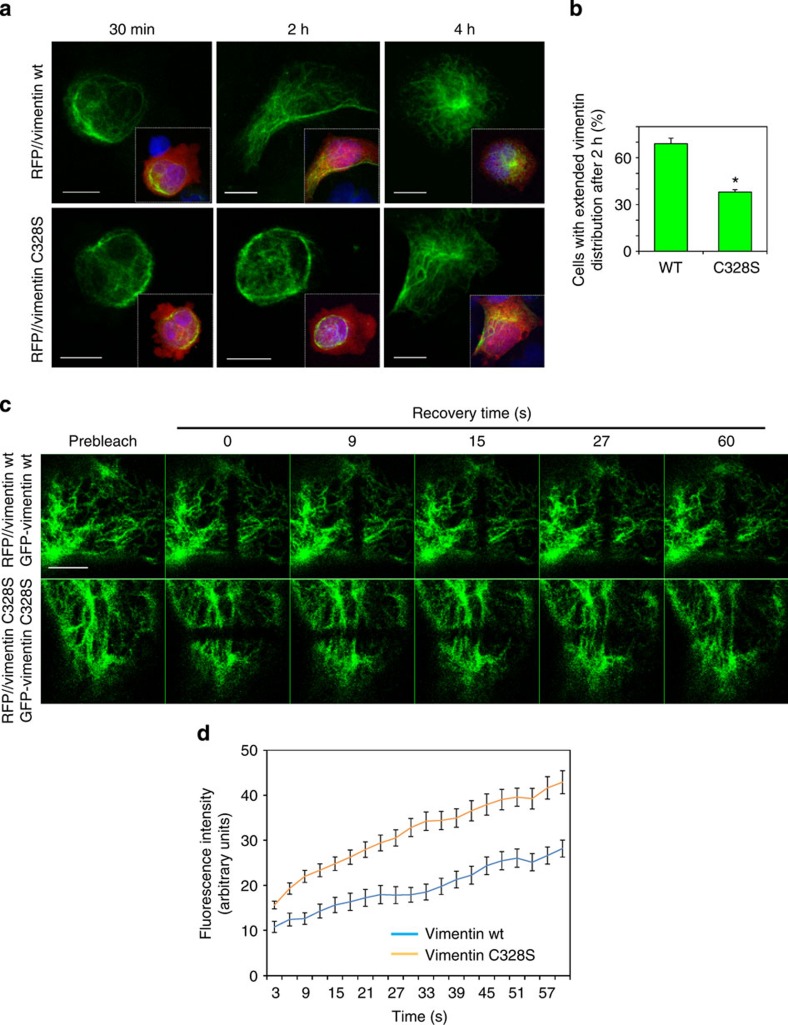
Dynamics of the vimentin network in cells expressing vimentin wt or C328S. (**a**) Expansion of the vimentin network after plating. SW13 cells stably transfected with RFP//vimentin wt or C328S were tripsinized, plated and fixed at the indicated times for IF of vimentin. Insets show RFP fluorescence (cytoplasm) and 4,6-diamidino-2-phenylindole staining (nuclei) to evidence the relative extension of the vimentin network. Scale bars, 10 μm. (**b**) The graph presents results at 2 h (mean±s.e.m., *n*=3, *P<0.05 by *t*-test). (**c**) FRAP assays of GFP-vimentin wt and C328S. SW13 cells were stably transfected with RFP//vimentin plus GFP-vimentin wt or C328S constructs. Selected images of fluorescence recovery after bleaching are shown. Scale bar, 5 μm. (**d**) Results of average fluorescence at each time point±s.e.m. (wt, *n*=26; C328S, *n*=48). Differences between values for vimentin wt and C328S were statistically significant at all time points by Student's *t*-test.

Our results suggest that C328 is not required for vimentin network formation under sustained growth conditions but may be important for its primary assembly. To test this, we monitored the expansion of the vimentin network after plating the cells on glass coverslips (Fig. 3a). Vimentin showed a perinuclear distribution upon plating, with filaments extending towards the cell periphery in a time-dependent fashion. Interestingly, this process was delayed in RFP//vimentin C328S-expressing cells, as clearly observed after 2 h (Fig. 3b). Nevertheless, after 4 h, most cells displayed an extended network. Overall cell spreading as monitored by the area of the cytoplasm (Fig. 3a, insets) was similar in wt and C328S-expressing cells.

Vimentin filaments assemble by both end-to-end annealing and may exchange subunits at any point along the filament length^26^,^28^. Subunit exchange at the mid-length of filaments was monitored by fluorescence recovery after photobleaching (FRAP) assays (Fig. 3c). The recovery time of filament fluorescence was shorter for C328S versus wt vimentin. This behaviour is clearly reflected by the increased slope and absolute fluorescence values reached by C328S vimentin filaments during the first stages of recovery (Fig. 3d). These observations suggest that subunit exchange is faster in C328S vimentin, whereas vimentin wt filaments appear more stable.

### Role of C328 in vimentin-mediated organelle positioning

**Figure 4:**
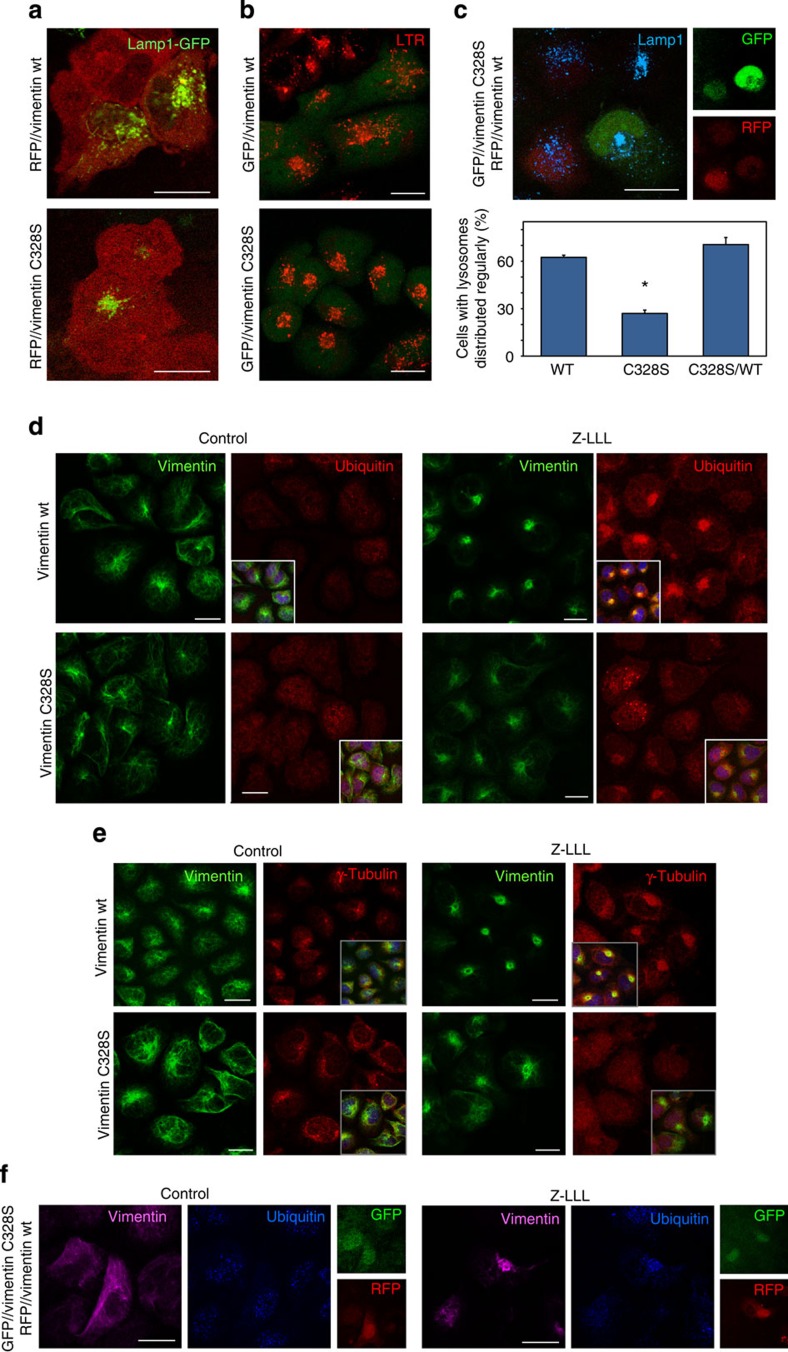
Effect of vimentin wt or C328S in lysosome positioning and aggresome formation. (**a**) SW13 cells were stably transfected with RFP//vimentin wt or C328S, transfected with Lamp1-GFP and visualized 48 h later by confocal microscopy. (**b**) Cells stably transfected with GFP//vimentin wt or C328S were stained with LTR. (**c**) Cells stably expressing GFP//vimentin C328S were transfected with RFP//vimentin wt (as shown in the right panels) and stained with anti-Lamp1. The graph shows the proportion of cells with evenly distributed lysosomes in the different conditions (mean±s.e.m., *n*=3, *P<0.05 by *t*-test versus vimentin wt). (**d**,**e**) Assessment of aggresome formation. Cells stably transfected with RFP//vimentin wt or C328S were treated with vehicle or Z-LLL, fixed and the indicated proteins were visualized by IF. Images shown are single sections to evidence co-localization of the structures of interest. Insets are overlay images depicting 4,6-diamidino-2-phenylindole staining; *n*≥3. (**f**) Cells transfected as in **c** showing the effect of Z-LLL in aggresome formation in cells expressing GFP//vimentin C328S, alone or together with RFP//vimentin wt, as depicted in the right panels. Scale bars, 20 μm.

Vimentin deficiency is associated with juxtanuclear accumulation of lysosomes^2^ (Supplementary Fig. 2a). Therefore, we explored the ability of wt or C328S vimentin to restore lysosome positioning in SW13 cells. First, cells stably transfected with RFP//vimentin wt or C328S were transiently transfected with Lamp1-GFP (Fig. 4a). In addition, cells stably transfected with GFP//vimentin wt or C328S were stained with Lysotracker Red (LTR; Fig. 4b). Both approaches revealed a more efficient restoration of lysosome positioning in vimentin wt than in C328S-expressing cells (Fig. 4c, graph).

Vimentin is involved in aggresome formation. When proteasomal degradation is impaired, misfolded proteins form cytoplasmic aggregates that migrate along microtubules and converge at the microtubule organizing center (mTOC) into larger structures known as aggresomes^45^. Aggresomes contain ubiquitinated proteins and γ-tubulin, surrounded by intermediate filaments forming cage-like structures^46^. Treatment of SW13 RFP//vimentin wt cells with the proteasome inhibitor Z-LLL (Z-Leu-Leu-Leu-al) elicited vimentin redistribution into large juxtanuclear coffer-like structures, co-localizing with ubiquitin (Fig. 4d) and γ-tubulin (Fig. 4e) accumulations. Interestingly, all these effects were attenuated in vimentin C328S-expressing cells (Fig. 4d,e, lower panels), which resembled non-transfected cells (Supplementary Fig. 2b). Therefore, C328 mutation affects vimentin efficacy in aggresome formation.

Both deficient lysosome positioning (assessed by Lamp1 IF) and aggresome formation in vimentin C328S-expressing cells were ameliorated by transfection of wt vimentin (Fig. 4c,f). Taken together, these results indicate that C328 is important for vimentin-dependent cellular processes.

### C328 is a sensor for electrophilic and oxidative stress

**Figure 5:**
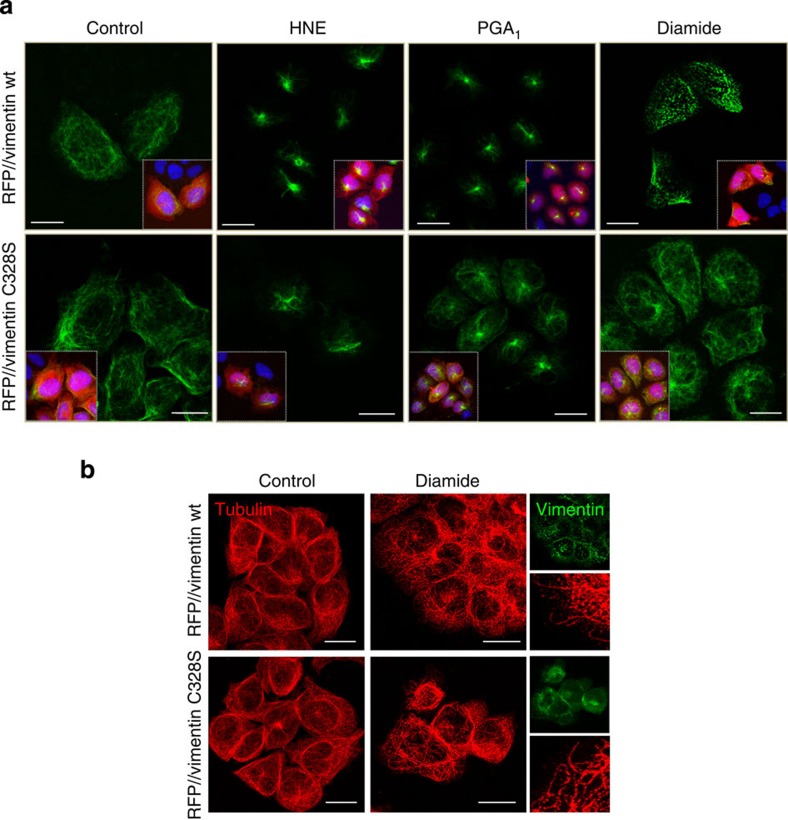
Importance of C328 in the response of the vimentin network to electrophilic and oxidative stress. SW13 cells stably transfected with RFP//vimentin wt or C328S were treated with 10 μM HNE for 4 h, 30 μM PGA_1_ for 20 h or 1 mM diamide for 20 min. (**a**) Vimentin network visualized by IF. Insets depict cytoplasmic RFP fluorescence and 4,6-diamidino-2-phenylindole nuclear staining; *n*≥3. (**b**) IF of tubulin. Insets show vimentin distribution and zoom-ins of areas of interest. Scale bars, 20 μm.

We next explored the effect of electrophilic and oxidative agents on cells expressing vimentin wt or C328S. Treatment with HNE induced the typical juxtanuclear condensation of vimentin in 70% of RFP//vimentin wt-expressing cells, but only in 33% of cells expressing vimentin C328S (Fig. 5a). Similarly, vimentin C328S-expressing cells were partially protected against PGA_1_-induced vimentin condensation (Fig. 5a). The oxidant diamide, which depletes reduced glutathione and induces vimentin glutathionylation^31^,^32^, elicited a drastic disassembly of filaments, which appeared in vimentin dots. Remarkably, this effect was abolished in vimentin C328S-expressing cells. Identical results were obtained with vimentin C328A (Supplementary Fig. 3). These results demonstrate that C328 is required for the reorganization of the vimentin network in response to electrophilic and oxidative stress and is essential for diamide-induced disassembly. The protective effect of the C328 mutation was specific for vimentin, since diamide induced a profound alteration of microtubules both in vimentin wt and C328S-expressing cells (Fig. 5b).

### C328 is involved in vimentin–vimentin interactions

**Figure 6:**
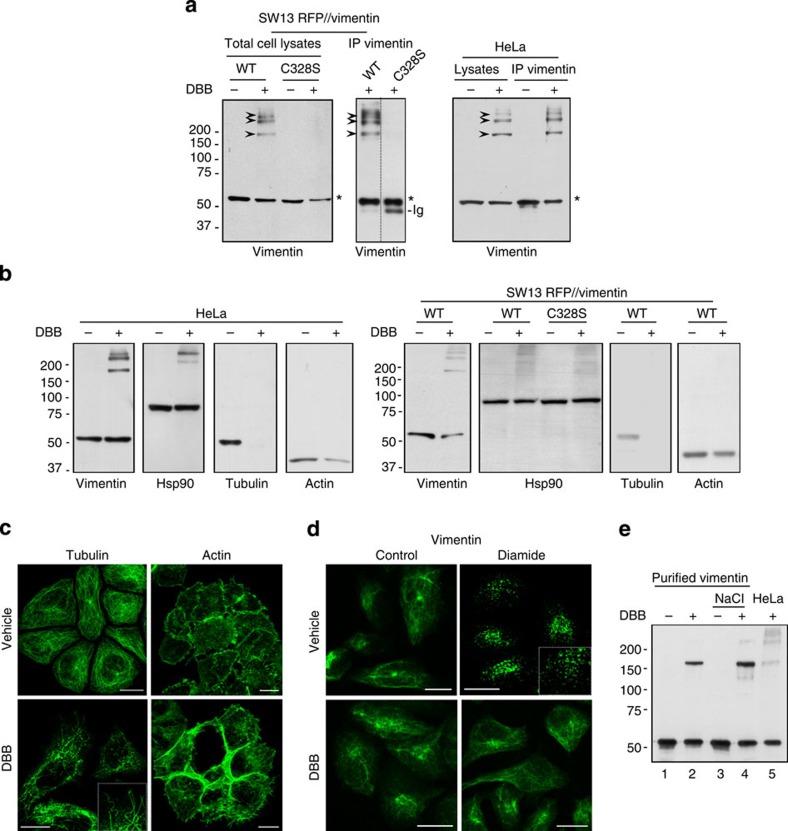
Crosslinking of vimentin by the bifunctional cysteine reagent DBB stabilizes the vimentin network. (**a**) DBB induces C328-dependent vimentin oligomerization in several cell types. SW13 stably transfected with RFP//vimentin wt or C328S, or HeLa cells were treated with vehicle or 100 μM DBB for 15 min, lysed and analysed by WB. Where indicated, vimentin was immunoprecipitated (IP) and analysed by WB. The vimentin monomer is marked by an asterisk and vimentin oligomers by arrowheads; Ig, immunoglobulin. (**b**) HeLa cells or SW13 RFP//vimentin cells were treated with DBB and the oligomerization of the proteins of interest analysed by WB; *n*≥3 for all oligomerization experiments. (**c**) Effect of DBB on the organization of various cytoskeletal structures. SW13 RFP//vimentin wt cells were treated with vehicle or DBB. Microtubules and actin distributions were visualized by IF and Phalloidin-Alexa568 staining, respectively. (**d**) SW13 RFP//vimentin wt cells were treated with vehicle or DBB for 15 min before addition of diamide. Vimentin was analysed by IF. Scale bars, 20 μm. (**e**) Induction of purified vimentin oligomerization by DBB. Soluble or polymerized (NaCl) vimentin was incubated with vehicle or 20 μM DBB and analysed by WB (*n*≥4). An aliquot of a HeLa cell lysate from DBB-treated cells was analysed in parallel to compare the apparent MW of the oligomers obtained.

The results above indicate that C328 mutation has two sides: it protects the vimentin cytoskeleton from disorganization by oxidants or electrophiles, but it impairs various basal vimentin functions. This suggests that the thiol group is involved in interactions important for the dynamics of the vimentin network. Therefore, we attempted detecting proteins with thiol groups in the proximity of C328. The bifunctional compound dibromobimane (DBB) irreversibly crosslinks vicinal thiol groups^47^,^48^. Treatment of SW13 cells expressing RFP//vimentin wt with DBB resulted in formation of several vimentin oligomers with apparent molecular weight of 170 kDa or higher (Fig. 6a), which did not appear in RFP//vimentin C328S-expressing cells, thus showing that their formation depends on the C328 thiol group. DBB induced similar vimentin-containing oligomers in cell types expressing endogenous vimentin, such as HeLa cells, accounting for 20–50% of the vimentin signal (Fig. 6a). These results indicate that in cells, vimentin C328 is in close proximity of other thiol groups. Immunoprecipitation and proteomic analysis of vimentin oligomers revealed the presence of several proteins, the most abundant of which, based on the frequency of the peptides observed, was vimentin (Supplementary Table 1). Other proteins identified included tubulin, Hsp90, actin or plectin, all of which appeared with 3–10 times less frequency. Western blot (WB) analysis showed a lack of correspondence between DBB-induced vimentin oligomeric species and the patterns observed for other proteins (Fig. 6b). In HeLa cells, DBB induced Hsp90 oligomerization but with a pattern different from vimentin oligomers. Moreover, in SW13 cells, DBB induced Hsp90 oligomerization independently of the presence of vimentin C328. DBB elicited a marked reduction in the detection of tubulin and a moderate reduction in the levels of monomeric actin. Consistent with these changes, both the tubulin and actin cytoskeletons suffered marked alterations upon DBB treatment (Fig. 6c). Tubulin appeared as distorted microtubules or punctate structures, whereas actin showed strong accumulations and stress fibres. In sharp contrast, the vimentin network was preserved in DBB-treated cells (Fig. 6d). These observations may indicate that, opposite to the disrupting effect on tubulin or actin, cysteine crosslinking of vimentin stabilizes filament structure. Therefore, we explored whether DBB protected the vimentin network from disruption by other agents. Pre-incubation of SW13 cells with DBB completely prevented diamide-induced vimentin disassembly (Fig. 6d), thus selectively preserving vimentin network architecture. A potential mechanism for this stabilizing effect could be the crosslinking of neighbouring vimentin subunits within filaments. To explore this, we assessed the capacity of DBB to crosslink vimentin *in vitro*. Incubation of vimentin with DBB led to the appearance of a major oligomeric species at 170 kDa (Fig. 6e), co-migrating with the oligomer observed in cells (compare lanes 2 and 5 in Fig. 6e). This electrophoretical behaviour is consistent with that of the previously characterized vimentin dimer^22^. Interestingly, DBB-induced vimentin crosslinking was more efficient with NaCl-polymerized vimentin (3.9±1.2-fold dimer in polymerized versus non-polymerized vimentin), suggesting that the disposition of the cysteine residues in vimentin filaments is more favourable for DBB-induced crosslinking than in soluble vimentin.

### Zinc interacts with vimentin and induces polymerization

**Figure 7:**
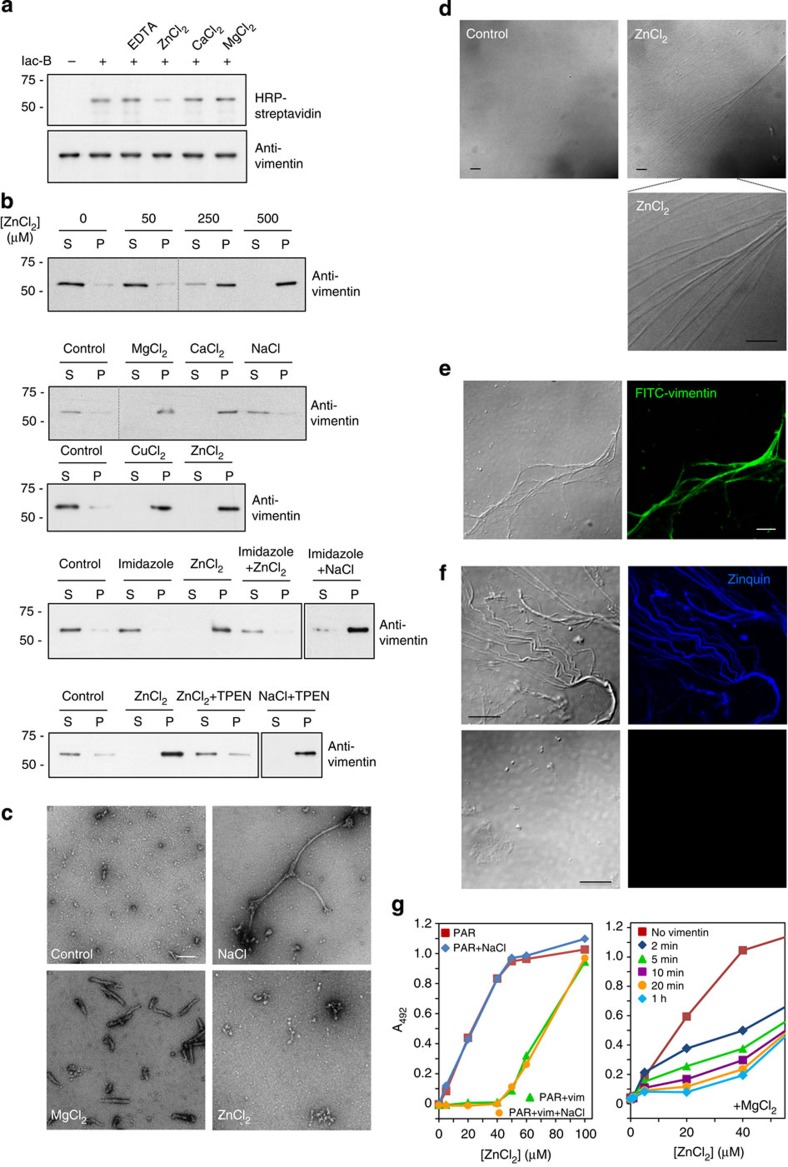
Interaction of vimentin with zinc. (**a**) Incubation with ZnCl_2_ selectively protects vimentin from modification by Iac-B. Polymerized vimentin was incubated with 10 mM EDTA or 500 μM ZnCl_2_, CaCl_2_ or MgCl_2_ before addition of 10 μM Iac-B. Incorporation of Iac-B and vimentin amounts were assessed as in Fig. 1. (**b**) Effect of zinc and other divalent cations on vimentin polymerization. Vimentin was incubated with the indicated concentrations of ZnCl_2_ or with 500 μM MgCl_2_, CaCl_2_, CuCl_2_, ZnCl_2_ or NaCl for 1 h at r.t., after which, soluble (S) and polymerized (P) vimentin were assessed as in Fig. 1. The effect of imidazole on vimentin polymerization induced by 500 μM ZnCl_2_ or 150 mM NaCl was evaluated as above. Reversion of ZnCl_2_-induced vimentin polymerization by TPEN was performed as described in Methods section; *n*≥3 for all polymerization assays. (**c**) Analysis of vimentin structures by electron microscopy (EM). Vimentin was incubated with 150 mM NaCl or 500 μM ZnCl_2_ for 1 h or with 2.5 mM MgCl_2_ o/n, before processing and visualization by EM at 50,000-fold magnification (*n*≥7). Scale bar, 100 nm. (**d**) Zinc induces vimentin assembly into optically detectable structures. Vimentin was incubated with 2.5 mM ZnCl_2_ at r.t. for 15 min and the structures formed visualized by optical microscopy (*n*=19). (**e**) Structures formed upon ZnCl_2_ treatment of FITC-labelled vimentin observed by fluorescence confocal microscopy. (**f**) Labelling of ZnCl_2_-induced vimentin filaments with Zinquin (upper panels) and loss of polymerization and Zinquin fluorescence upon TPEN treatment (lower panels). Scale bars, 10 μm, *n*≥3. (**g**) Competition assays between vimentin and PAR for zinc binding. Left panel, mixtures containing 100 μM PAR and 1 μM soluble or NaCl-polymerized vimentin were incubated with increasing concentrations of ZnCl_2_ and the formation of the ZnPAR_2_ complex was monitored by the absorbance at 492 nm. Right panel, effect of magnesium on the ability of vimentin to sequester zinc. Assay mixtures containing PAR or PAR plus vimentin were preincubated with MgCl_2_, after which ZnCl_2_ was added and the ZnPAR_2_ complex was determined at the indicated time points (representative from four assays).

The presence of closely positioned cysteine residues in vimentin filaments could serve a regulatory and/or structural role. Since we did not obtain evidence for vimentin disulfide bond formation and the distance spanned by DBB should be longer than that of a disulfide bond, we hypothesized that cysteine residues could interact through metal coordination. Interestingly, incubation of vimentin in the presence of ZnCl_2_, but not CaCl_2_ or MgCl_2_, clearly reduced labelling by Iac-B (Fig. 7a), thus indicating that zinc may interact with the vimentin cysteine. EDTA, used to remove potentially conjugated metals, was without effect, as expected, since vimentin is purified under strong denaturing conditions. Remarkably, micromolar concentrations of ZnCl_2_ induced vimentin polymerization *in vitro* (Fig. 7b). Other divalent cations, including Mg^2+^, Cu^2+^ and Ca^2+^ induced vimentin polymerization, whereas, NaCl did not induce full polymerization at this concentration. Imidazole, which contributes to zinc coordination in proteins through the imidazole moiety of histidine residues and may act as a zinc chelator, effectively prevented zinc-induced polymerization, whereas NaCl-induced polymerization was not affected. Finally, zinc-induced polymerization was reversed by addition of the zinc chelator *N*,*N*,*N*′,*N*′-tetrakis(2-pyridylmethyl)ethylenediamine (TPEN).

We next explored the morphology of the vimentin polymers by electron microscopy (Fig. 7c). Non-polymerized vimentin showed small particles or elongated structures compatible with unit-length filaments^49^, whereas 150 mM NaCl induced the formation of the typical 10-nm-wide filaments. MgCl_2_ induced homogeneous filaments with a diameter similar to NaCl-induced filaments but consistently shorter. Unfortunately, the structures observed after incubation with ZnCl_2_ were scarce and highly heterogeneous. To evaluate whether formation of aggregates could cause these effects, ZnCl_2_-induced vimentin polymerization was carried out on a glass slide for direct visualization by optical microscopy. Strikingly, this approach showed the presence of exceptionally long and thick filament bundles (Fig. 7d). Use of fluorescein isothiocyanate (FITC)-labelled vimentin allowed the visualization of zinc-induced fluorescent filaments (Fig. 7e). Pretreatment with Iac reduced the formation of these structures (Supplementary Fig. 4). Moreover, zinc-induced filaments were intensely stained by the fluorescent zinc-probe Zinquin, which can detect both free and protein-bound zinc^50^; conversely, addition of TPEN disorganized the filaments and blunted Zinquin fluorescence (Fig. 7f). In addition, vimentin avidly bound zinc as evidenced by its competition with the high affinity, zinc-specific dye 4-(2-pyridylazo)-resorcinol (PAR), which forms a coloured ZnPAR_2_ complex (*K*_d_=3.0 × 10^−13^; ref. 51). Both soluble and NaCl-polymerized vimentin effectively competed for zinc binding up to a zinc:vimentin ratio of ∼40:1 (Fig. 7g, left panel). This indicated that zinc binding by vimentin was supra-stoichiometric, potentially due to multiple electrostatic interactions. Indeed, magnesium at concentrations in the range of those present intracellularly, hampered zinc sequestration by vimentin, as evidenced by the higher level of ZnPAR_2_ complex detected (Fig. 7g, right panel). However, this effect was reduced with time, suggesting complex, dynamic interactions between vimentin and divalent cations.

### Effect of zinc on the cellular vimentin network

**Figure 8:**
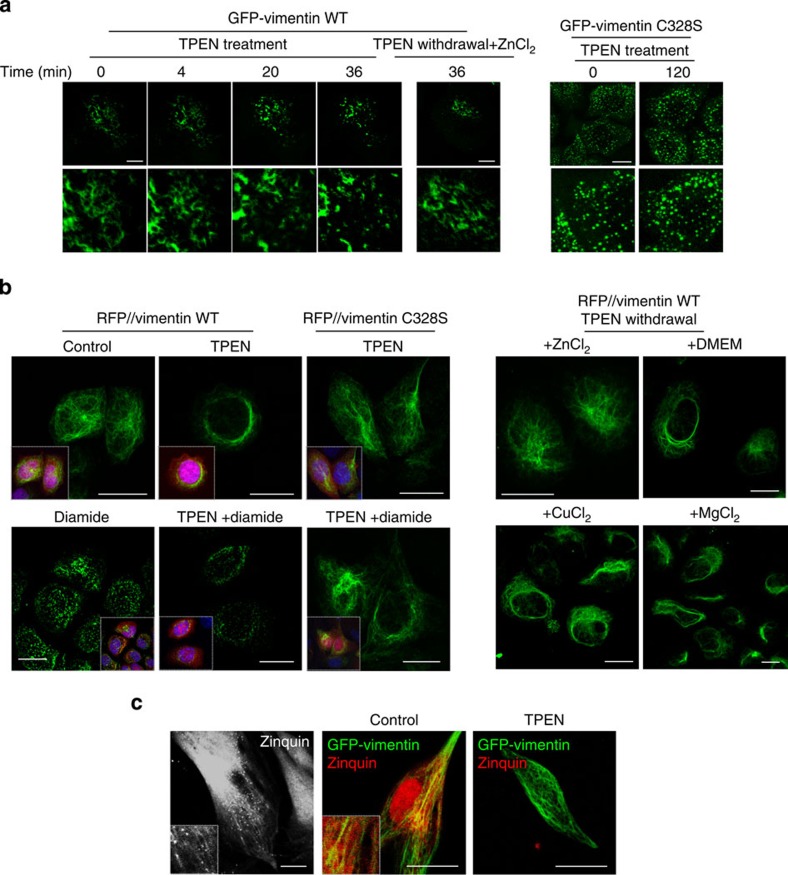
Modulation of the cellular vimentin network by zinc. (**a**) SW13 cells stably transfected with GFP-vimentin wt or C328S were treated with TPEN for the indicated times and monitored by live confocal microscopy. Lower panels show higher magnification. For recovery, TPEN was withdrawn and 30 μM ZnCl_2_ was added for an additional 36 min. Scale bars, 10 μm. (**b**) SW13 cells stably transfected with RFP//vimentin wt or C328S were treated with vehicle or TPEN for 6 h, or TPEN followed by diamide, and vimentin was visualized by IF; *n*=3. Scale bars, 20 μm. (**c**) SW13 cells stably transfected with RFP//vimentin wt were treated with TPEN as in **b**. After 6 h, the medium was replaced by fresh serum-free medium alone (DMEM) or supplemented with the indicated salts at 30 μM, and cells were allowed to recover o/n before vimentin IF; *n*=3 (**d**) Distribution of Zinquin in human fibroblasts, non-transfected (left panel) or co-transfected with GFP-vimentin, before (central panel) or after treatment with TPEN (right panel); *n*≥4.

The above results demonstrate an avid interaction between vimentin and zinc *in vitro*, resulting in the assembly of multifilament structures. However, due to its dependence on the presence of other cations, the relevance of this interaction needs to be assessed in the cellular milieu. Therefore, we explored the effect of zinc on vimentin organization in cells. Treatment of SW13 cells expressing GFP-vimentin wt with TPEN led to the rapid disassembly of GFP-vimentin short filaments into dots (Fig. 8a). Remarkably, removal of TPEN and addition of ZnCl_2_ allowed reappearance of short filaments within 1 h. TPEN did not alter the dotted distribution of GFP-vimentin C328S (Fig. 8a, right panels). In RFP//vimentin wt-expressing cells, which possess a full vimentin network, TPEN induced a marked reorganization of vimentin into shorter filaments, forming a perinuclear or juxtanuclear ‘cap' (Fig. 8b). Moreover, pretreatment with TPEN potentiated diamide-induced vimentin network disruption, leading to a decrease in vimentin IF signal. TPEN effects required C328, since filaments formed by C328S vimentin were not disrupted by either TPEN or TPEN plus diamide. TPEN-induced vimentin alterations were selectively reversed by removal of the chelator and addition of ZnCl_2_, at concentrations in the range of those found in plasma^52^,^53^, (Fig. 8b, right panels). Removal of TPEN alone or combined with addition of other divalent cations, including Mg^2+^ and Cu^2+^, did not revert the effect of zinc depletion (Fig. 8b, right panels). Thus, cellular zinc availability may function as a reversible switch controlling vimentin dynamics and susceptibility to oxidants through its interaction with C328, thus playing a key role in vimentin function.

To substantiate the vimentin–zinc interaction in cells, we stained primary human fibroblasts, which endogenously express high vimentin levels, with Zinquin. This probe showed a predominantly diffuse distribution, with some punctate accumulations and filamentous structures. Transfection with GFP-vimentin wt to light up the vimentin network revealed that Zinquin decorated extended segments of vimentin filaments. Treatment with TPEN abrogated Zinquin staining and co-localization with vimentin, thus suggesting that zinc binds to vimentin filaments in cells.

### Modulation of vimentin function by zinc in pathophysiology

We finally explored the importance of zinc availability for vimentin network structure and response to stress in a pathophysiologically relevant model. For this, we used primary fibroblasts from an acrodermatitis enteropathica (AE) patient (GM02814A cells), which display decreased zinc uptake and content^54^.

**Figure 9:**
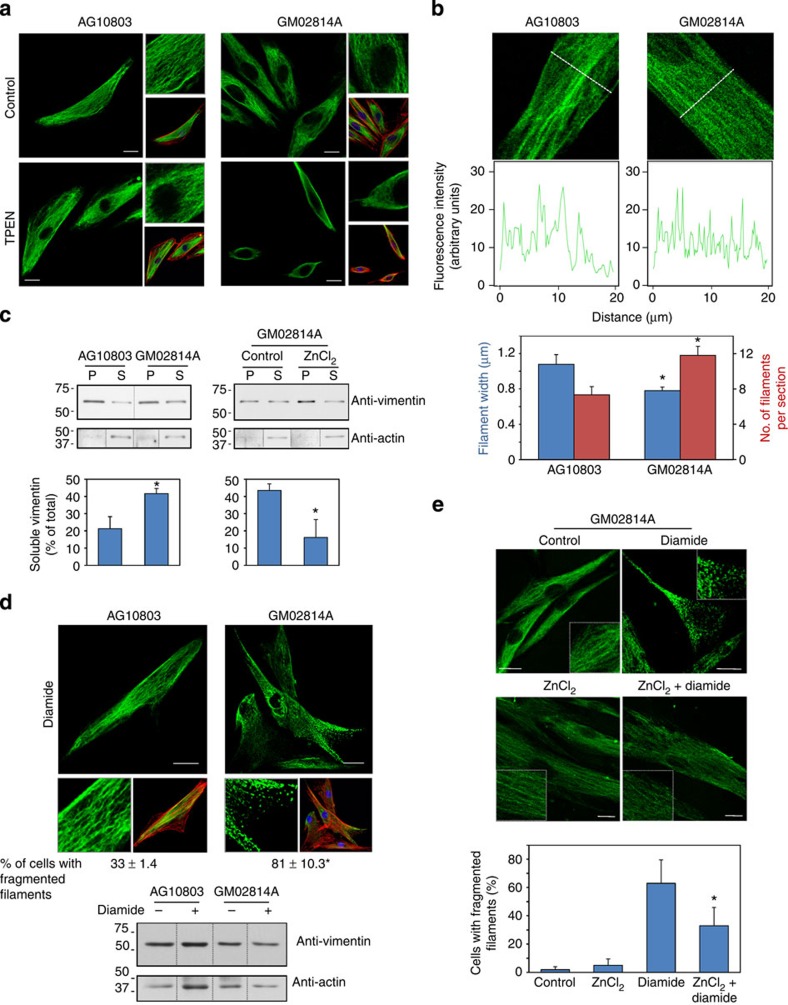
Vimentin distribution in a pathophysiological situation of defective zinc transport. (**a**) Effect of TPEN in control and AE fibroblasts. Primary fibroblasts from a control subject (AG10803 cells) or from an AE patient (GM02814A cells) were cultured for 4 h with vehicle or TPEN. Vimentin and actin were visualized by IF and Phalloidin-Alexa 568 staining, respectively. Nuclei were stained with 4,6-diamidino-2-phenylindole. Right panels show zoom-ins and overlays of vimentin (green), actin (red) and nuclei (blue); *n*≥3. (**b**) Vimentin filament width and the number of filaments per transversal cellular section were evaluated by image analysis. Lower panels show fluorescence intensity profiles along the dotted lines. Graphed values are mean±s.e.m. of 44 and 98 filaments in control and AE fibroblasts, respectively; *P<0.05 by *t*-test (**c**) Proportion of soluble and pelletable vimentin in control and AE fibroblasts (left panels) and effect of supplementation of AE fibroblasts with 30 μM ZnCl_2_ for 1 h (right panels); *n*=3, *P<0.05 by *t*-test. (**d**) Effect of diamide on the vimentin network in AG10803 and GM02814A cells. Cells were treated with diamide, fixed and stained as above. Lower panels show zoom-ins and overlays of vimentin, actin and nuclei. Percentages of cells with fragmented filaments (dots) are shown (*n*=3, *P<0.05 by *t*-test). In the lower panel, vimentin and actin levels were assessed by WB. (**e**) Protective effect of zinc on diamide-induced disruption of the vimentin cytoskeleton. GM02814A cells were preincubated for 1 h in serum-free medium alone (control) or supplemented with 30 μM ZnCl_2_ before diamide treatment. The proportion of cells presenting vimentin in dots is shown; (*n*=3) data are mean±s.e.m; *P<0.05 by *t*-test. Scale bars, 20 μm.

Fibroblasts from control subjects (AG10803 cells) showed a uniform longitudinal distribution of vimentin filaments (Fig. 9a), which, in fibroblasts from AE patients displayed a more variable orientation and lower density in the perinuclear region. Interestingly, zinc depletion of control fibroblasts induced a vimentin distribution similar to that of AE fibroblasts, whereas the effect on these latter cells was more marked, with vimentin condensation at the subcortical area, and scarcer and diffuse filaments at the cell centre.

To explore the structural features of the vimentin network potentially related to zinc availability, we measured filament width in control and AE fibroblasts. Control cells displayed thicker vimentin filaments, as well as fewer filaments per transversal cell section, suggesting that zinc may favour lateral association (Fig. 9b). Short-term zinc supplementation of AE cells induced a normalizing, although not significant, trend of these parameters (Supplementary Fig. 5). Consistent with a role of zinc in vimentin assembly, the proportion of soluble vimentin was higher in AE fibroblasts and was significantly reduced by zinc supplementation (Fig. 9c). AE cells were also more susceptible to oxidants. Diamide elicited a partial fragmentation of vimentin filaments in control fibroblasts but extensive disassembly in AE cells (Fig. 9d). These effects were not associated with changes in vimentin levels or electrophoretic mobility (Fig. 9d, lower panel). Zinc pretreatment of AE cells attenuated the effect of diamide, clearly preserving filament linear integrity (Fig. 9e).

Taken together, our results show that zinc availability is important for vimentin network structure under pathophysiological conditions. Moreover, they support a protective role of zinc against vimentin reorganization or disassembly by oxidants at least in part through interaction with C328.

## Discussion

**Figure 10:**
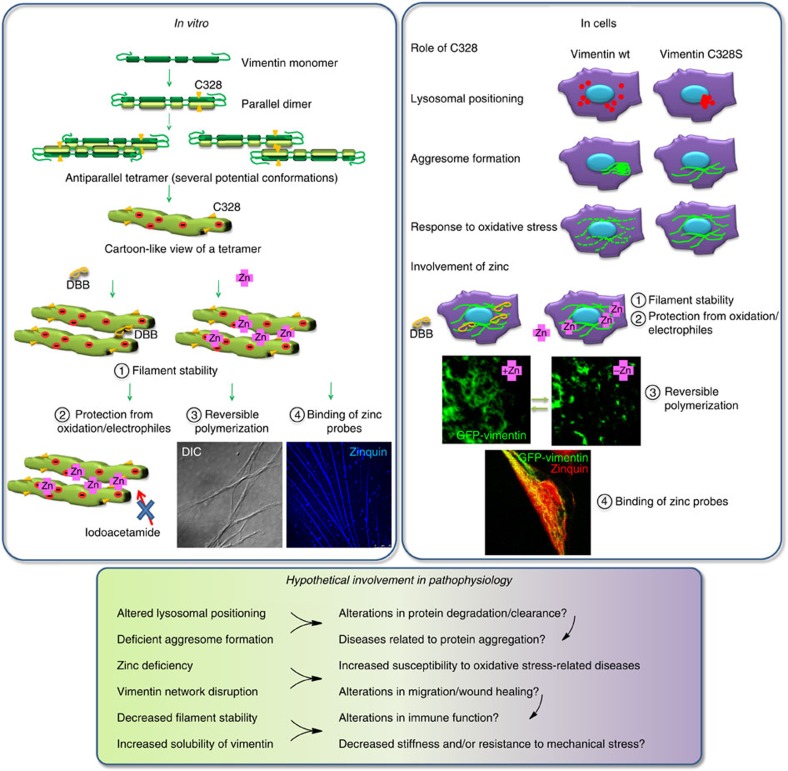
Schematic overview of the roles of C328 and zinc in vimentin filament assembly and their potential pathophysiological implications.

Vimentin plays a key role in cell biology and pathophysiology far beyond its structural function: it not only contributes to cell adhesion, migration and organelle positioning, and facilitates wound repair but is also involved in epithelial–mesenchymal transition and microorganism invasion. Here we show that the highly conserved vimentin cysteine residue is important for the organization and function of the vimentin network, since its mutation compromises vimentin performance in network expansion, correct lysosomal positioning and aggresome formation, as well as the response of the vimentin network to oxidative and electrophilic stress. Moreover, C328 is involved in interaction with zinc, thus playing a role in vimentin network stability and dynamics (summarized in Fig. 10).

Although C328 has been identified in proteomic studies as a target for several oxidative modifications^34^,^41^, elucidation of its cellular role has been hampered by the use of cells expressing endogenous vimentin or GFP fusion constructs, which may result in anomalous distribution. As shown here, whereas both GFP-vimentin wt and the C328 mutant incorporate into preformed vimentin networks, the GFP tag interferes with filament elongation in vimentin-deficient cells^55^, and amplifies the functional defect of C328 mutation. In turn, although non-tagged vimentin C328S was able to form an extended network, it did not rescue the phenotype of vimentin-deficient cells.

The essential role of C328 for vimentin reorganization in response to electrophilic agents and oxidants is best exemplified by diamide, which induced disassembly of vimentin filaments in virtually all vimentin wt-expressing cells but was without effect in cells expressing C328 mutants. A potential interaction of the thiol group of C328 with other proteins or molecules could reconcile its requirement for optimal vimentin performance with the protective role of its mutation against oxidative vimentin damage. This was supported by the effect of the bifunctional thiol crosslinker DBB, which induced the formation of vimentin oligomers appearing as dimers under denaturing conditions, and selectively stabilized the vimentin network eliciting a full protection against diamide. Thus, DBB appeared to crosslink vimentin filaments in their ‘native' conformation, while drastically altering other cytoskeletal networks.

Although several models for vimentin assembly have been proposed, the modes of interaction in cells are not known. Our results indicate that cysteine residues in filaments may exist at a distance of 3–6 Å, which is that spanned by DBB, therefore longer than the 2.05-Å distance existing between sulfur atoms in disulfide bonds. Our primary hypothesis to account for this distance was the presence of a metal, such as zinc, bridging the cysteine residues^56^. Coordination of zinc would confer stability to the interaction between vimentin wt oligomers, as observed in FRAP assays, in contrast with the more active exchange of subunits taking place on vimentin C328S filaments. Moreover, binding of zinc could provide an explanation for the dual role of C328 in vimentin function, favouring optimal alignment of vimentin oligomers, while protecting the cysteine from oxidation or modification with bulky moieties, which could induce filament disruption. C328 crosslinking with DBB could then stabilize the vimentin network by mimicking this optimal alignment.

We have evidenced the vimentin–zinc interaction both *in vitro* and in cells. *In vitro*, zinc protects vimentin from alkylation. Moreover, to the best of our knowledge, our results constitute the first demonstration of the induction of vimentin polymerization by micromolar zinc concentrations, leading to the formation of Zinquin-positive structures, orders of magnitude larger than single vimentin filaments, which are disrupted by the zinc chelator, TPEN. Furthermore, PAR-competition studies showed incorporation of zinc into vimentin with high affinity, which precluded detection of free zinc by PAR up to 40 μM. Thus, zinc could interact with vimentin at multiple sites, as described for other divalent cations *in vitro*^57^, likely through electrostatic interactions involving its multiple charged residues. The fact that millimolar magnesium slowed down zinc capture by vimentin, points to a possible exchange between both divalent cations on the protein, with preferential zinc binding. Notably, zinc binds to vimentin at concentrations that do not induce polymerization (low micromolar), indicating that it can bind to soluble vimentin species, which may range from monomers to octamers^58^.

In cells, pharmacological or genetic alteration of zinc levels has drastic consequences on vimentin organization. Pharmacological effects are most striking in SW13 cells, in which zinc chelation/supplementation can induce several rounds of disassembly and reassembly of GFP-vimentin short filaments. Genetic alteration is observed in the autosomal recessive disease AE, caused by mutations in the zinc-specific transporter *SLC39A4/ZIP4*, which mediates intestinal and cellular absortion of zinc^59^,^60^. This leads to low zinc serum levels and symptoms including dermatitis, diarrhoea and alopecia^61^, as well as to zinc-uptake defects and low cellular zinc content^54^,^62^. AE fibroblasts showed both basal alteration of the vimentin network and increased susceptibility to zinc depletion and oxidative stress. Some of these features were recapitulated by zinc depletion of control fibroblasts. In turn, zinc supplementation of AE fibroblasts rapidly improved the ratio between polymerized and soluble vimentin and attenuated the drastic fragmentation of the vimentin network by diamide. Zinc protective effects were short-termed, pointing to a direct effect on vimentin association and/or oxidation state. Indeed, although zinc binding to other cytoskeletal structures cannot be excluded, our results show for the first time that vimentin filaments can be decorated by Zinquin staining in cells, supporting a direct interaction.

Remarkably, vimentin C328 mutants are refractory to zinc modulation, supporting C328 involvement in zinc interaction. This is reinforced by the observation that magnesium, copper and calcium did not protect C328 from alkylation and were unable to revert the cellular effects of zinc depletion. Therefore, zinc could interact with vimentin with great affinity, both through electrostatic forces, depending on the ionic environment, and through C328. The relative contribution of these two potential mechanisms will be the subject of further studies.

Besides a direct role of zinc in vimentin polymerization, stability, and protection from oxidants, indirect effects should also be considered, above all in the long term, including zinc roles in redox homeostasis^63^ and gene expression, through interaction with zinc-finger containing transcription factors.

Our results show that either acute or chronic zinc deficiency disturbs vimentin function and susceptibility to stress, potentially contributing to pathophysiology. Zinc deficiency is extremely frequent (>20% of the total population), with symptoms ranging from dermatitis, diarrhoea and alopecia to cognitive alterations, higher susceptibility to infections, impaired wound healing, growth retardation^64^ and cardiac oxidative damage^65^. Prevalence of insufficient dietary zinc uptake is particularly high in developing countries, as well as in the elderly, even in industrialized countries^64^. Vimentin is expressed in many different cell types in which a zinc–vimentin interaction could be pathophysiologically relevant, including immune, endothelial, undifferentiated muscle and neuronal cells. Therefore, the potential involvement of impaired vimentin function in the alterations associated with zinc deficiency deserves further investigation.

In summary, our results show that C328 of vimentin serves a dual role as sensor for oxidants and electrophiles, allowing the vimentin network to rapidly respond to various stress situations, and as a key residue in vimentin stability and dynamics conceivably through zinc binding. These results will shed light on the biology of vimentin and related cytoskeletal structures, thus opening novel perspectives for the understanding of varied biological processes.

## Methods

### Reagents

HNE, 15-deoxy-Δ^12^,^14^-prostaglandin J_2_ (15d-PGJ_2_), prostaglandin A_1_ (PGA_1_) and their biotinylated derivatives were from Cayman Chemical. Diamide, DBB, Iac-B, TPEN, Z-LLL o MG132, Zinquin acid and Zinquin ethyl ester, anti-actin, anti-tubulin and anti-vimentin antibodies were obtained from Sigma. Monoclonal anti-Hsp90 (sc-7947), anti-Lamp1 (sc-20011) anti-vimentin V9 antibody (sc-6260) and its Alexa488, Alexa405 and agarose conjugates were obtained from Santa Cruz Biotechnology. Purified recombinant Syrian hamster vimentin (accession number AH001833) was from Cytoskeleton, Inc. Anti-HNE Michael adducts were obtained from Calbiochem. The monoclonal anti-tubulin P1C3 antibody was the generous gift of Dr Isabel Barasoaín (Centro de Investigaciones Biológicas, CSIC, Madrid, Spain). LTR, phalloidin-Alexa468 and phalloidin-Alexa568 were from Molecular Probes. Pefablock protease inhibitor was from Roche.

### Cell culture and treatments

The following cell cultures were obtained from the National Institute of General Medical Sciences (NIGMS) Human Genetic Cell Repository at the Coriell Institute for Medical Research (Candem, NJ): Human primary fibroblasts from control subjects (AG10803) and from patients with AE (ONIM entry 201100, cell line GM02814A), and were manipulated according to the instructions of the supplier. The study was conducted according to the Declaration of Helsinki principles and was approved by the Commission of Bioethics and Biosafety of Centro de Investigaciones Biológicas and by the Bioethics Committee of Consejo Superior de Investigaciones Científicas (Spain). SW13/cl.2 vimentin-deficient cells were the generous gift of Dr A. Sarria^43^ and were cultured in DMEM supplemented with 10% (vol/vol) fetal bovine serum and antibiotics (100 U ml^−1^ penicillin and 100 μg ml^−1^ streptomycin). Rat mesangial cells were obtained from rat kidneys using a technique of graded sieving^40^ and cultured in RPMI1640 with supplements as above. HeLa cells were from the American Type Cell Culture Collection. For treatments, cells were cultured in the absence of serum. Treatments with Z-LLL for proteasome inhibition were carried out at 200 nM for 20 h. Diamide was added for 20 min at 1 mM, where indicated. TPEN was used for zinc depletion in cells at 10 μM for the indicated times.

### Plasmids and transfections

Lamp1-GFP was the generous gift of Professor J. Lippincott-Schwartz (National Institutes of Health). The GFP-vimentin wt and GFP-vimentin C328S constructs have been previously described^40^. From these plasmids, vimentin wt and C328S were subcloned into the EcoRI and BamHI sites of pIRES2-AcGFP1 and pIRES2 DsRed-Express2 bicistronic plasmids, obtained from Clontech, to yield constructs that express the fluorescent protein and untagged vimentin as separate proteins. These constructs will be referred to as GFP//vimentin wt or C328S and RFP//vimentin wt or C328S throughout the manuscript. This strategy ensures the monitorization of cells expressing transfected vimentin (GFP or RFP positive, respectively) to distinguish them from the few SW13 cells that can undergo spontaneous reversion of vimentin expression. The plasmids GFP-vimentin C328A and RFP//vimentin C328A were generated from GFP-vimentin and RFP//vimentin wt, respectively, by using the Quickchange XL mutagenesis kit from Stratagene with oligonucleotides: 5′-GGTGCAGTCCCTCACCGCTGAAGTGGATGCCC-3′ and its complementary reverse. For transient transfections, cells in p35 plates were transfected with 1 μg of DNA using Lipofectamine 2,000 (Invitrogen). For co-transfection assays, 1 μg of bicistronic plasmids coding for untagged vimentin wt or C328S and RFP for identification of transfected cells (RFP//vimentin), along with 0.2 μg of the GFP-vimentin fusion constructs were used. For generation of stably transfected cells, selection was carried out by culture in the presence of 500 μg ml^−1^ of G-418 (Gibco) for at least 3 weeks.

### *In vitro* assays for vimentin modification and polymerization

For modification by various electrophiles, vimentin at 5 μM in 5 mM PIPES (pH 7.0) and 0.1 mM dithiothreitol (DTT) was incubated in the presence of the indicated compounds dissolved in dimethylsulfoxide. In these assays, either soluble or polymerized vimentin was used. In the case of incorporation of biotinylated reagents, biotin detection was achieved by incubation of blots with horseradish peroxidase (HRP)-streptavidin and enhanced chemiluminiscence (ECL). HNE incorporation was detected with an anti-HNE Michael adducts antibody.

For polymerization, vimentin was incubated in the presence of 5 mM PIPES (pH 7.0) and 150 mM NaCl, for 5–20 min at 37 °C. In other assays, polymerization was induced by the addition of various salts of divalent cations, as indicated. The effect of imidazole on ZnCl_2_-induced polymerization was assessed by co-incubation with 1 M imidazole. For reversion of ZnCl_2_-induced polymerization, vimentin polymerized by incubation with 500 μM ZnCl_2_ for 1 h was subsequently incubated with 1 mM TPEN for 30 min. In all cases, soluble (S) and polymerized (P) vimentin were separated by ultracentrifugation at 100,000*g* for 30 min at 4 °C, after which, aliquots of the supernatant, containing soluble vimentin, and of the pellets, resuspended in Laemmli buffer, were analysed by SDS–PAGE and WB.

Crosslinking assays were performed using vimentin preincubated in the absence or presence of 150 mM NaCl, at 3 μM, by incubation in the presence of 20 μM DBB for 1 h at 37 °C. Aliquots from the incubations were separated on 7.5% SDS–polyacrylamide gels and analysed by WB.

For assessment of zinc binding, we used a modification of a recently described procedure for the detection of zinc-binding proteins, which is based on the reduction of the incorporation of a cysteine-reactive probe in the presence of this metal^66^. Briefly, purified vimentin was polymerized with 150 mM NaCl for 10 min at 37 °C before incubation for 1 h at r.t. with 10 mM EDTA or 500 μM ZnCl_2_, CaCl_2_ or MgCl_2_. Subsequently, 10 μM Iac-B was added for additional 30 min. Incorporation of Iac-B was assessed by SDS–PAGE and blot with HRP-streptavidin and vimentin amount by WB. In this assay, incubation with EDTA serves as a control to remove potential divalent cations bound to the protein. In this case, since vimentin is purified under strong denaturing conditions, it is not expected that the protein retain conjugated metals.

### Visualization of vimentin structures by optical microscopy

A 5-μl aliquot of vimentin at 10 μM in 5 mM PIPES (pH 7.0) and 0.1 mM DTT was placed on a glass slide, and 5 μl of 5 mM ZnCl_2_ solution was added and mixed with the pipette tip, after which, a coverslip was placed on top of the solution and the sample was visualized on a SP2 confocal microscope using the differential interference contrast mode for image acquisition. Assays were also carried out at 500 μM zinc with similar results. For preparation of fluorescent vimentin, vimentin at 17 μM was incubated for 15 min at r.t. in the presence of a 10-fold molar excess of FITC, after which, excess FITC was removed by gel filtration on Zeba desalting microspin columns (Thermo Scientific) following the instructions of the manufacturer. FITC–vimentin was mixed with unmodified vimentin in a 1:4 proportion and processed as above for visualization by optical microscopy. For Zinquin staining, 1 mM Zinquin acid was added to the vimentin–ZnCl_2_ mixture before placing the coverslip. In some assays, the mixture of vimentin–ZnCl_2_, incubated for polymerization as above, was subsequently treated with 2 mM TPEN before addition of Zinquin. Zinquin fluorescence was detected by excitation with a ultraviolet laser at 351 and 364 nm, and emission between 450 and 520 nm was acquired.

### Electron microscopy

Vimentin at 3 μM was incubated in the presence of various salts at r.t. Incubation mixtures were fixed with 0.25% glutaraldehyde immediately before application onto carbon-coated copper–palladium grids. After blotting off excess sample, grids were washed with polymerization buffer and negatively stained with 2% uranyl acetate for 90 s. Samples were observed in a JEOL JEM-1230 electron microscope operating at 100 kV equipped with a CMOS TVIPS TemCam-F416 digital camera and obtained at 50 K magnification.

### Immunofluorescence

After the various treatments, cells were fixed with 4% paraformaldehyde for 25 min at r.t. Cells were permeabilized with 0.1% Triton X-100 in PBS, blocked by incubation in 1% bovine serum albumin in PBS (blocking solution) and incubated with anti-vimentin-Alexa488 or the indicated primary antibodies at 1:200 dilution in the same solution. When necessary, secondary antibodies were used at 1:200 dilution in blocking solution. Cells were counterstained with 4,6-diamidino-2-phenylindole at 3 μg ml^−1^ in PBS for 15 min. For actin visualization, cells were stained with Phalloidin-Alexa488 or Alexa568, following the instructions of the manufacturer. Lysosomes were stained with LTR by incubating cells with 25 nM LTR for 30 min at 37 °C (ref. 67). When coverslips were used, they were mounted with Fluorsave from Calbiochem.

### Confocal microscopy

Cells were visualized on Leica SP2 or SP5 microscopes. Individual sections were taken every 0.5 μm with a numerical aperture of × 63, and overall projections are shown, unless otherwise stated.

For FRAP assays, the Leica SP5 confocal microscope equipped with a thermostatized chamber at 37 °C was used. Briefly, a prebleach image was taken, after which three pulses of 488 nm laser power were applied to bleach an area of 18 × 1.5 μm. Post-bleach images were acquired every 3 s for 10 min using software from Leica. For analysis of fluorescence recovery, the intensity in defined points of bleached filaments was measured at the different time points with Fiji ImageJ software^68^ and expressed in arbitrary units to plot recovery graphs. A total of 20 FRAP assays were carried out per experimental condition; 26 wt and 48 C328S filaments were monitored in total.

For estimation of vimentin filament thickness, cross-sections of single confocal microscopy planes of fibroblasts stained for vimentin by IF were plotted to obtain profiles using Fiji ImageJ software^68^. The width of peaks with intensity >50% maximum intensity was measured individually, as well as the number of filaments per cell section.

For visualization of intracellular zinc, cells grown on glass-bottom dishes were incubated with 25 μM Zinquin ethyl ester in Hank's Balanced Salt Solution (HBSS) and observed on a Leica SP2 microscope using a thermostatized chamber at 37 °C. Zinquin fluorescence was detected with the excitation and emission parameters specified above.

### Vimentin network expansion assay

SW13 cells stably transfected with RFP//vimentin wt or C328S were trypsinized and plated on glass coverslips. At the indicated time points after plating, cells were fixed and processed for IF with anti-vimentin antibody. The position of nuclei was obtained by 4,6-diamidino-2-phenylindole staining and the area occupied by the cytoplasm was revealed by RFP fluorescence. The pattern of the vimentin network was assessed by independent observers in a blind manner to evaluate the proportion of cells with vimentin filaments circumscribed to the perinuclear area or extending towards the periphery of the cells. A minimum of 100 cells per assay were monitored.

### Vimentin solubility assays

The proportion of soluble and insoluble vimentin in cells was determined by cell extraction with a high salt, detergent buffer (20 mM Tris-HCl, pH 7.4, 600 mM NaCl, 0.5% Triton X-100, 0.1 mM sodium orthovanadate and protease inhibitors (2 μg ml^−1^ each of leupeptin, aprotinin and trypsin inhibitor, and 1.3 mM Pefablock) and separation by centrifugation at 12,000*g* for 10 min at 4 °C (ref. 69). Equal volumes from soluble and insoluble fractions were analysed by SDS–PAGE and WB, and disparities in protein loading were monitored by WB anti-actin. Non-saturated exposures from three independent experiments were analysed by image scanning, and the proportion of soluble vimentin was calculated with respect to total vimentin.

### WB and immunoprecipitation

For SDS–PAGE, cells were lysed in 20 mM Tris-HCl, pH 7.5, 0.1 mM EDTA, 0.1 mM EGTA, 0.1 mM β-mercaptoethanol, containing 0.5% SDS, 0.1 mM sodium orthovanadate and protease inhibitors (2 μg ml^−1^ each of leupeptin, aprotinin and trypsin inhibitor, and 1.3 mM Pefablock). Aliquots containing 20 μg of protein were separated on SDS–polyacrylamide gels of the adequate percentage of polyacrylamide, as indicated. Gels were transferred to Immobilon-P membranes (Millipore) using the three buffer system recommended by the manufacturer. Blots were incubated with the antibodies against the proteins of interest (typically at 1:500 dilution) followed by secondary antibodies (HRP conjugated) at 1:2,000 dilution, and immunoreactive bands were detected with ECL (GE Healthcare). In some instances, lanes from the same gel have been cropped for presentation. This is indicated by dotted lines in figures and uncropped scans of blots are presented in Supplementary Fig. 6.

For vimentin immunoprecipitation, cells were lysed in 50 mM Tris-HCl, pH 7.5, 0.1 mM DTT and 0.5% SDS containing protease inhibitors. Lysates were diluted 1:5 in the same buffer in which SDS was substituted by 1% NP-40 and incubated overnight with anti-vimentin V9 agarose-conjugated antibody. Immunoprecipitates were washed four times with PBS and eluted by incubation at 95 °C for 5 min with Laemmli buffer.

### Proteomic analysis

Immunoprecipitates obtained from 400 μg of lysates of DBB-treated HeLa cells with the anti-vimentin agarose-conjugated antibody were separated by SDS–PAGE. Gels were stained with Sypro Ruby and were visualized under ultraviolet light. The region of the gel corresponding to the DBB-induced oligomers detected by WB was excised and destained using 50 mM ammonium bicarbonate/50% acetonitrile (ACN), reduced with 10 mM DTT for 30 min at r.t., alkylated with 55 mM iodoacetic acid in the dark for 30 min at r.t. and digested with 12.5 ng μl^−1^ trypsin in 50 mM ammonium bicarbonate, overnight (o/n) at 30 °C. Peptides were extracted with ACN and 5% trifluoroacetic acid (TFA) and cleaned using ZipTips (0.6 μl C18 resin, Millipore).

Peptides were resuspended in 0.1% formic acid/2% ACN (buffer A) and analysed in an LTQ Orbitrap Velos (Thermo Scientific) in the positive ion mode, coupled to a nanoEasy HPLC (Proxeon). Peptides were first trapped onto a C18-A1 ASY-Column 2 cm precolumn (Thermo Scientific), and then eluted onto a Biosphere C18 column (C18, inner diameter 75 μm, 15 cm long, 3 μm particle size; NanoSeparations) and separated using a 70-min gradient from 0 to 35% buffer B (buffer B: 0.1% formic acid in ACN) at a flow rate of 250 nl min^−1^. Full-scan mass spectra (*m/z* 300–1,700) were acquired in the Orbitrap with a target value of 1,000,000 at a resolution of 30,000 at *m/z* 400, and the 15 most intense ions were selected for collision-induced dissociation fragmentation in the LTQ with a target value of 10,000 and normalized collision energy of 35%. Acquired spectra were searched against human UniProt database (090513) using Sequest search engine through Proteome Discoverer (version 1.4.1.14, Thermo). As for the search parameters, precursor and fragment mass tolerance were set to 10 p.p.m. and 0.8 Da, respectively. Carbamidomethylation of cysteines was set as a fixed modification, and oxidation of methionines was set as a dynamic modification. Two missed cleavages were allowed. Identified peptides were validated using Percolator algorithm with a *q* value threshold of 0.01 and those with a high confidence level were accepted. The number of peptides from any given identified protein matching these criteria is referred to as peptide spectrum match.

### PAR-competition assays

Assays for the competition between PAR and vimentin were based in the procedure described by Koch *et al*.^51^, with the following modifications: PAR was used at 100 μM final concentration and vimentin at 1 μM. The assay was carried out in 5 mM PIPES, pH 7.0, at r.t. Assays were started by the addition of ZnCl_2_ solution to give final concentrations between 1 and 100 μM. The formation of the ZnPAR_2_ complex (*K*_d_ of ZnPAR_2_, 3 × 10^−13^; ref. 51) was determined photometrically at 492 nm. No significant changes in absorbance were observed during the time of the assay (typically 10 min). In some assays, vimentin was preincubated with 20 mM MgCl_2_ for 50 min, after which ZnCl_2_ was added. In this case, changes in absorbance with time were followed. PAR samples received an equivalent amount of MgCl_2_, which did not affect colour development.

### Statistical analysis

All experiments were repeated at least three times. For experiments involving visual inspection of morphological features, preparations were evaluated by two independent observers in a blind fashion, and, unless otherwise stated, at least 50 cells were monitored per experimental condition. All results are presented as average values±s.e.m. Statistical differences were evaluated by the Student's *t*-test and were considered significant when *P*<0.05, which is denoted in graphs by an asterisk.

## Additional information

**How to cite this article:** Pérez-Sala, D. *et al*. Vimentin filament organization and stress sensing depend on its single cysteine residue and zinc binding. *Nat. Commun.* 6:7287 doi: 10.1038/ncomms8287 (2015).

## Supplementary Material

Supplementary Information Supplementary Figures 1-6 and Supplementary Table 1.
