# Supplementary material for: Vimentin filament organization and stress sensing depend on its single cysteine residue and zinc binding
Source: Nat Commun. 2015 Jun 2;6:7287. doi: 10.1038/ncomms8287 (PMC4458873; doi:10.1038/ncomms8287)
Supplement: Supplementary Information — Supplementary Figures 1-6 and Supplementary Table 1. [file ncomms8287-s1.pdf]

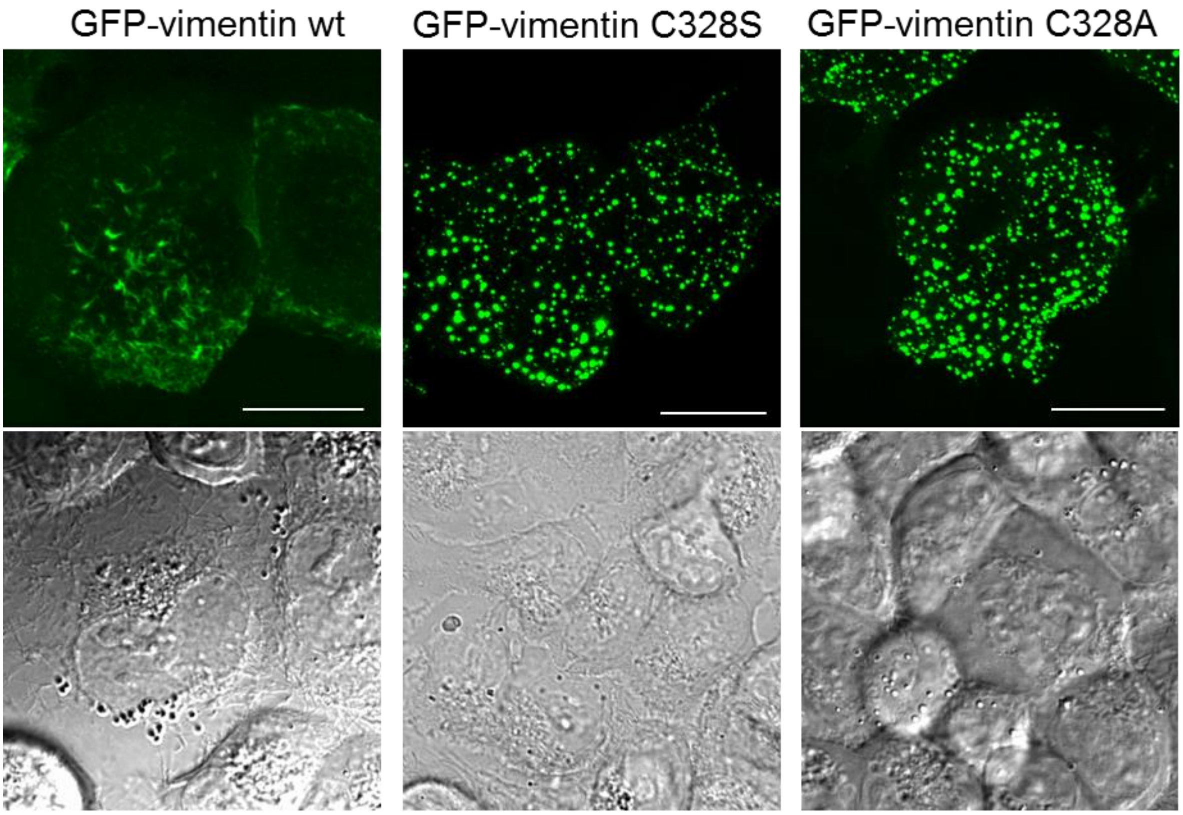

Supplementary Figure 1. Pattern of GFP-vimentin in SW13 cells. Cells were transfected with the indicated constructs as in Fig. 2B; n=3. Fluorescence and DIC images of live cells are shown. Bars, 20 μm.

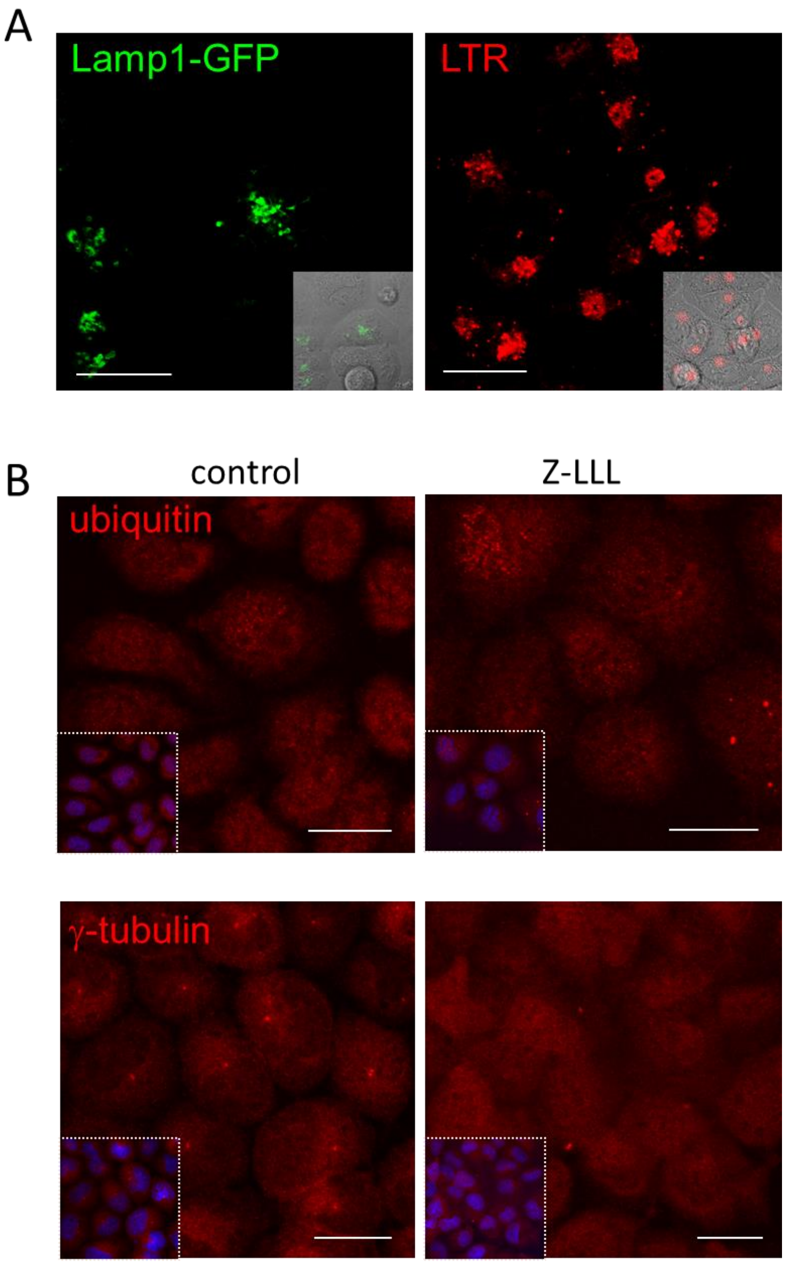

Supplementary Figure 2. Phenotype of untransfected SW13 cells. (A) Distribution of lysosomes as evidenced by the position of Lamp1-GFP and LTR-positive compartments. Insets show overlays with DIC images. (B) Distribution of ubiquitin and γ-tubulin in control cells and cells treated with Z-LLL as in Figure 4D and E. Insets show overlays with DAPI staining. Results are representative of at least three experiments. Bars, 20 μm.

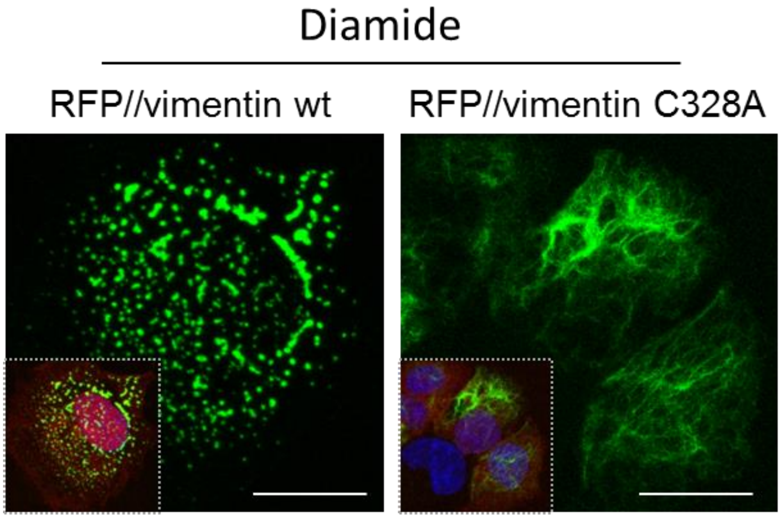

Supplementary Figure 3. Vimentin C328A is resistant to the effect of diamide. Cells were treated as in Fig. 5A. Shown is the vimentin IF. Insets show DAPI staining and RFP fluorescence; n=3. Bars, 20 μm.

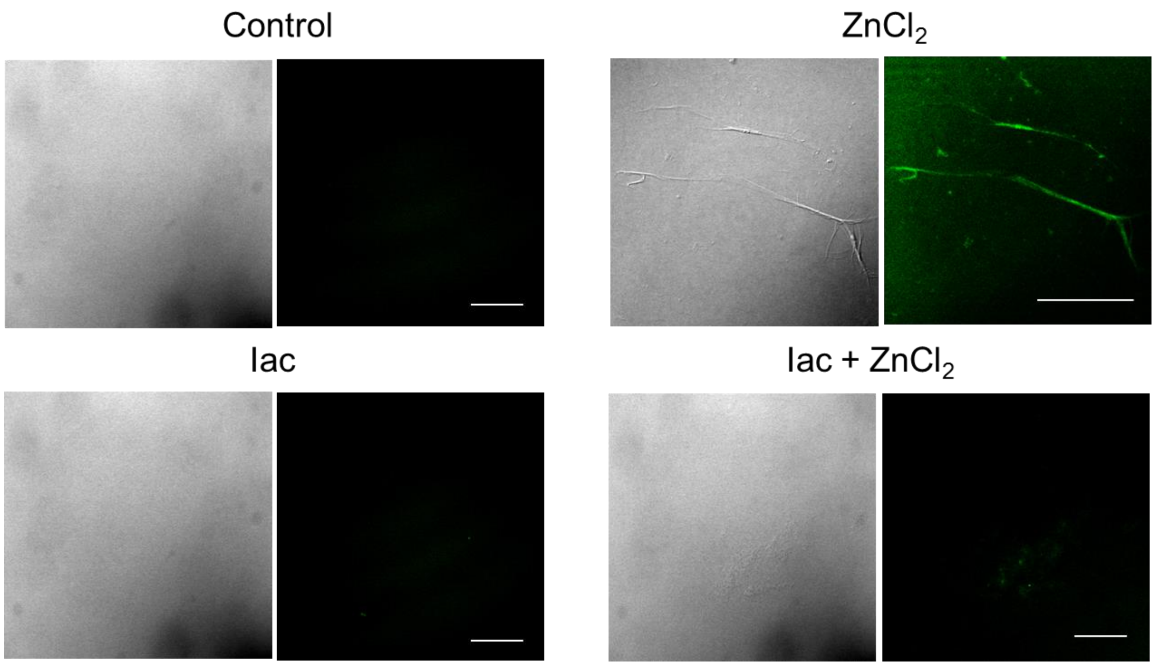

Supplementary Figure 4. Iodoacetamide blocks formation of FITC-vimentin structures. FITC-vimentin was incubated with $ZnCl_2$ before or after alkylation with iodoacetamide. Samples were processed as in Fig. 7E. Shown are DIC (left panels) and fluorescence (right panel) images for every condition. Results are representative from three assays. Bars, 30 μm.

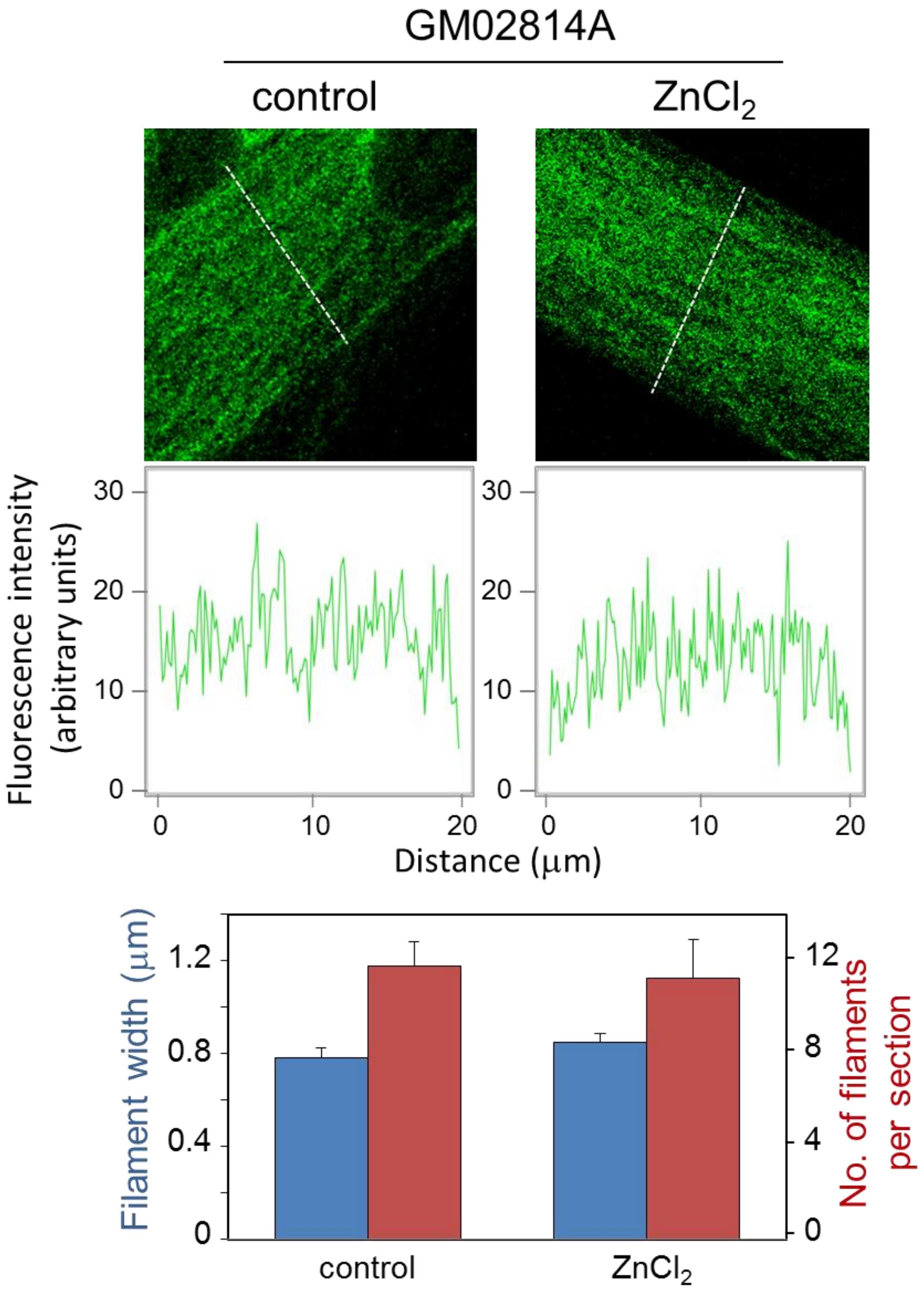

Supplementary Figure 5. Effect of zinc supplementation on filament width and number of filaments per transversal cellular section in AE fibroblasts (GM02814A cells). Graphed values are mean±S.E.M. of 178 and 200 filaments in control and $ZnCl_2$-treated fibroblasts, respectively.

Supplementary Figure 6. Full blots for figures in the main text.

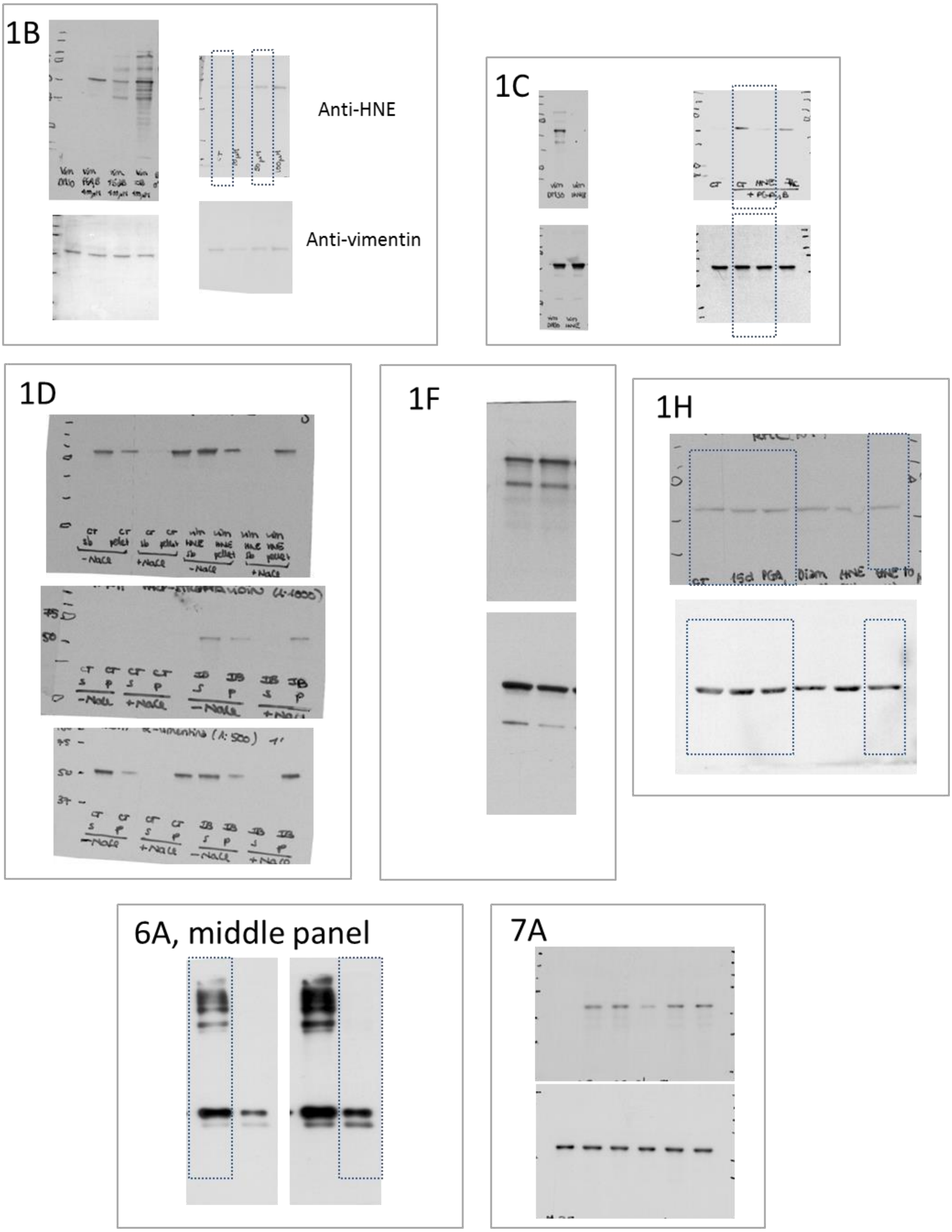

Supplementary Figure 6. Full blots for figures in the main text, continued.

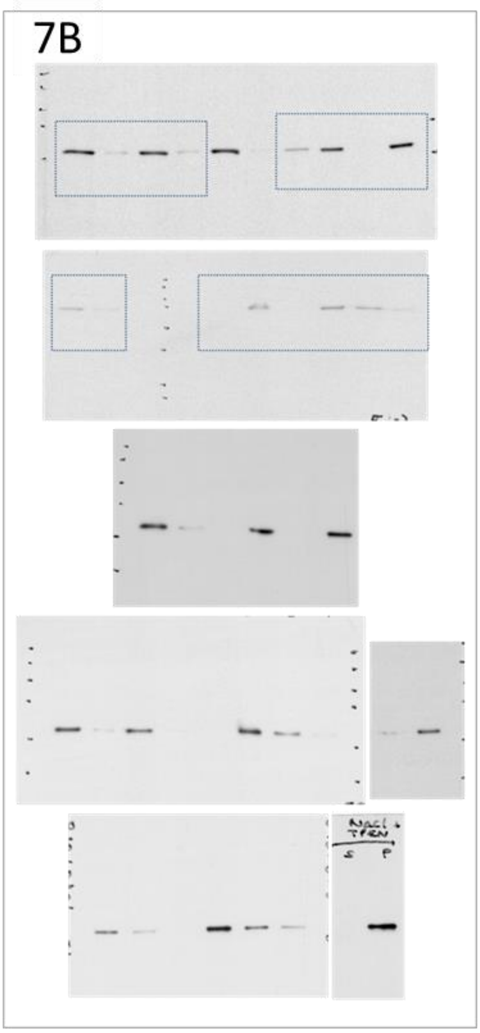

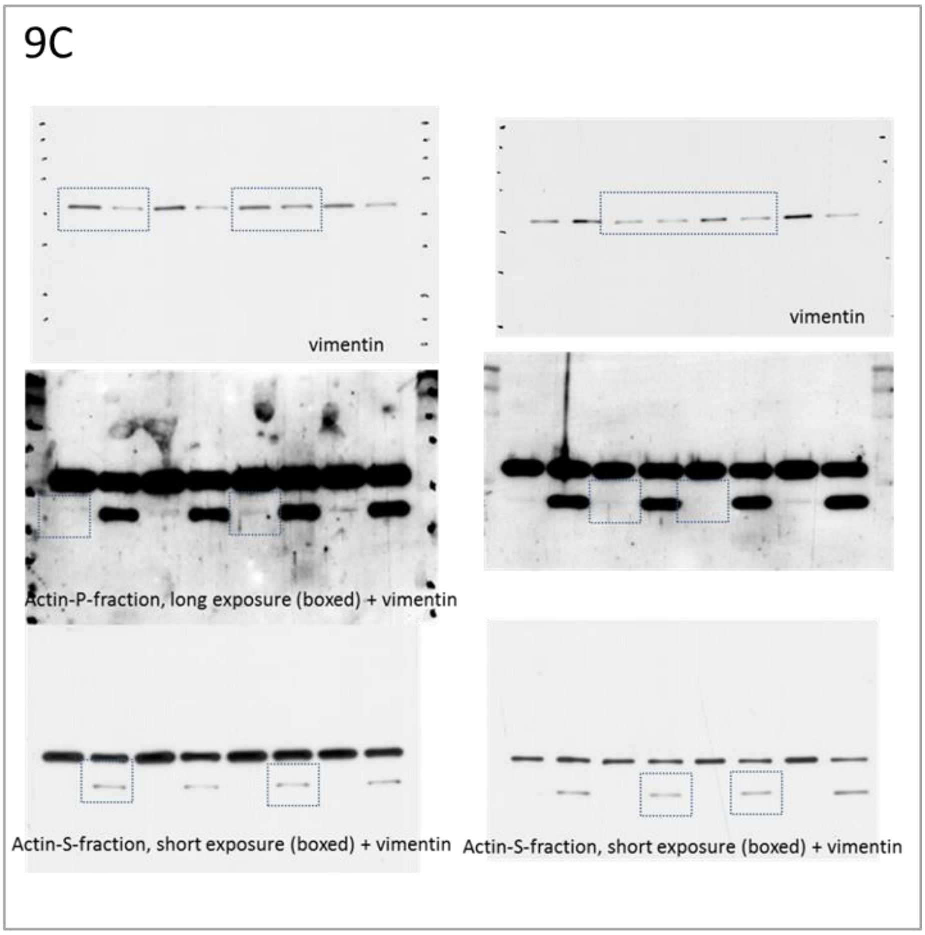

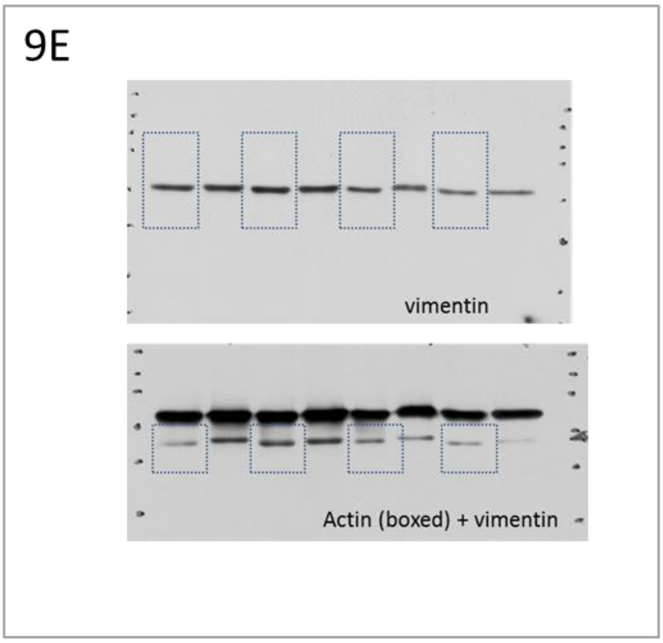

Supplementary Table 1. Proteins identified by proteomic analysis of immunoprecipitated vimentin oligomers shown in Figure 6.

	Accession number	Coverage (%)	Unique peptides	PSM	Protein grouping score	MW (Kda)
vimentin	P08670	58.58	27	91	30	53.6
Plectin (isoform 7)	Q15149-7	3.17	13	13	12	512.3
Neuroblast diff. assoc. protein	Q09666	12.72	19	28	1	628.7
Hsp90-beta	P08238	12.98	8	10	17	83.2
Tubulin-beta chain	Q5JP53	17.14	2	10	24	47.7
Actin (fragment)	I3L1U9	28.04	4	7	29	23.8
Hsp70 (fragment)	E9PN25	29.55	3	5	18	14.6
eIF5A	P63241	30.52	2	6	5	16.8

PSM, peptide spectrum match.
